# The Rice *ILI2* Locus Is a Bidirectional Target of the African *Xanthomonas oryzae* pv. *oryzae* Major Transcription Activator-like Effector TalC but Does Not Contribute to Disease Susceptibility

**DOI:** 10.3390/ijms23105559

**Published:** 2022-05-16

**Authors:** Hinda Doucouré, Florence Auguy, Servane Blanvillain-Baufumé, Sandrine Fabre, Marc Gabriel, Emilie Thomas, Fleur Dambreville, Coline Sciallano, Boris Szurek, Ousmane Koita, Valérie Verdier, Sébastien Cunnac

**Affiliations:** 1 LBMA, Faculté des Sciences et Techniques, University des Sciences Techniques et Technologiques, Bamako E 3206, Mali; doucourehinda@yahoo.fr (H.D.); okoita@icermali.org (O.K.); 2 PHIM Plant Health Institute, University Montpellier, IRD, CIRAD, INRAE, Institut Agro, 34398 Montpellier, France; florence.auguy@ird.fr (F.A.); servane.baufume@cirad.fr (S.B.-B.); sandrine.fabre@cirad.fr (S.F.); marc.gabriel@curie.fr (M.G.); emilie.thomas@ird.fr (E.T.); fleur.dambreville@gmail.com (F.D.); coline.sciallano@ird.fr (C.S.); boris.szurek@ird.fr (B.S.); valerie.verdier@ird.fr (V.V.)

**Keywords:** rice, *Xanthomonas oryzae*, TAL effector, disease

## Abstract

*Xanthomonas oryzae* pv. *oryzae* (*Xoo*) strains that cause bacterial leaf blight (BLB) limit rice (*Oryza sativa*) production and require breeding more resistant varieties. Transcription activator-like effectors (TALEs) activate transcription to promote leaf colonization by binding to specific plant host DNA sequences termed effector binding elements (EBEs). *Xoo* major TALEs universally target susceptibility genes of the SWEET transporter family. TALE-unresponsive alleles of clade III *OsSWEET* susceptibility gene promoter created with genome editing confer broad resistance on Asian *Xoo* strains. African *Xoo* strains rely primarily on the major TALE TalC, which targets *OsSWEET14*. Although the virulence of a *talC* mutant strain is severely impaired, abrogating *OsSWEET14* induction with genome editing does not confer equivalent resistance on African *Xoo*. To address this contradiction, we postulated the existence of a TalC target susceptibility gene redundant with *OsSWEET14*. Bioinformatics analysis identified a rice locus named ATAC composed of the *INCREASED LEAF INCLINATION 2* (*ILI2*) gene and a putative lncRNA that are shown to be bidirectionally upregulated in a TalC-dependent fashion. Gain-of-function approaches with designer TALEs inducing ATAC sequences did not complement the virulence of a *Xoo* strain defective for *SWEET* gene activation. While editing the TalC EBE at the ATAC loci compromised TalC-mediated induction, multiplex edited lines with mutations at the *OsSWEET14* and ATAC loci remained essentially susceptible to African *Xoo* strains. Overall, this work indicates that ATAC is a probable TalC off-target locus but nonetheless documents the first example of divergent transcription activation by a native TALE during infection.

## 1. Introduction

Bacterial leaf blight (BLB) is a widespread rice disease caused by *Xanthomonas oryzae* pv. *oryzae* (*Xoo*) [1]. The significant economic impact of this disease on rice cultivation [2] has prompted breeding commercial varieties for enhanced resistance traits by conventional introgression of resistance loci from the rice germplasm [3,4]. Recently, pioneering work [5] and the development of straightforward genome editing tools have, however, sparked a wave of reports on the engineering of BLB resistance in elite varieties [6,7,8,9,10,11]. In this strategy, the modified rice genomic sequences correspond to effector binding elements (EBEs), the DNA sequence recognized by a translocated bacterial virulence protein of the transcription activator-like effectors (TALEs) family. Editing a relevant EBE thus renders the altered genotype refractory to TALE-mediated BLB susceptibility gene induction and confers disease resistance.

Upon injection into the plant host cell via the molecular syringe of the bacterial type III secretion system (T3SS), TALEs enter the nucleus and promote the transcription of their target locus. TALEs were initially thought to induce transcription in a single direction by activating the synthesis of a protein-coding mRNA molecule on the same strand and 40–60 bp downstream of their target EBE [12,13,14]. However, this does not seem to be universal because the transcription of sequences on the opposite strand and upstream of the EBE was recently reported to occur in a couple of specific TALE–EBE combinations [15,16]. EBE sequence recognition is mediated by the central domain of the effector that wraps around the major groove of the genomic DNA and makes specific contacts with bases of this sequence. These interactions involve repeat variable diresidues (RVDs) corresponding to positions 12 and 13 of otherwise-conserved ~34 amino acid repeats that make up the central domain. In a colinear manner, amino acid 13 from successive repeats along the TALE central domain sequence recognizes a cognate nucleobase along the EBE strand. Subsequent to its discovery [17,18], the RVD–nucleotide association code has been relatively well characterized and prominent RVDs, such as NI, NG, HD, and NN, were shown to recognize A, T, C, and G/A, respectively [19]. As a general rule and with the exception of RipTALs [20], EBEs start with a T that is not recognized by a canonical repeat but rather by a structurally related helical bundle or a cryptic repeat located before the central domain [21,22,23]. Because this feature contributes to DNA recognition, it is ordinarily referred to as “repeat 0” and the corresponding location of the recognition site as “position 0.”

With a few notable exceptions, including the transcription elongation factor *OsTFIIAγ1* and the b-ZIP transcription factor *OsTFX1*, which were shown to be induced by TALE effectors from Philippine *Xoo* strains [24] or the African-*Xoo*-specific target *OsERF#123* [25], in rice, susceptibility genes exploited by *Xoo* major TALEs, which were thus edited for resistance engineering, belong to clade III of the *OsSWEET* gene family [26]. How their rice membranous sugar transporter products favor parasitism is still an unsettled issue, but they may support bacterial metabolism by releasing sucrose into the apoplast [27]. Virtually all pathogenic *Xoo* strains harbor at least one TALE inducing one of three *OsSWEET* clade III homologs. For instance, PthXo1 from the Philippine strain PXO99A [28] targets *OsSWEET11*, while PthXo2 variants from Asian Xoo target *OsSWEET13* [8,29]. Probably as a result of convergent evolution, overlapping or distinct EBEs have been identified on the *OsSWEET14* promoter. The cognate TALEs AvrXa7 and PthXo3 originate from Asian Xoo strains [30]. Others, namely TalC and TalF, have been described in African *Xoo* strains [26,31].

Both functional characterization and long read sequencing of the African *Xoo* TALE repertoires revealed that TalC is highly conserved and found in all analyzed strains. A large share of African strains also possesses the redundant TalF TALE [8]. Some strains, such as BAI3 [25] and MAI68 [32], contain a TalF variant that is inactive on the *OsSWEET14* promoter and, therefore, rely solely on TalC for *OsSWEET14* induction.

The genetic divide observed in phylogenetic studies between *Xoo* strains of the Asian and African lineages [33,34] also appears in the prospects of engineering BLB resistance with modified, TALE-unresponsive, *OsSWEET* promoter alleles. While this strategy possesses an immense potential for the broad control of Asian *Xoo* strains, as previously demonstrated [8,9], the situation appears less straightforward regarding African *Xoo* bacteria. This is because the dependency of African *Xoo* strains on the SWEET susceptibility component for host colonization differs from that of the Asian strains analyzed so far. Initial clade III *OsSWEET* promoter editing approaches to engineer resistance to African strains were unsuccessful [35]: While *OsSWEET14* induction upon African *Xoo* BAI3 inoculations was abrogated in TalC-EBE-edited rice lines, those lines, in two different Japonica rice backgrounds, remained essentially susceptible to this Burkinabe strain. A subsequent study obtained equivalent results when challenging Kitaake lines with the Malian MAI68 strain [32]. More recent findings align with this conclusion and provide additional insight. For instance, a single *OsSWEET14* knockout event in Kitaake background exhibits moderately reduced susceptibility (about half the lesion length of wild-type plants) to AXO1947, BAI3, or MAI1 African strains [7]. In contrast, an *OsSWEET14* knockout mutant in rice cultivar Zhonghua 11 appears to completely abolish lesions caused by AXO1947 [11], suggesting that other polymorphic loci may epistatically influence the disease outcome depending on the rice genotype. The susceptibility of quintuple-EBE-edited lines from Oliva et al. [8] is again modestly decreased when challenged with the AXO1947 strain. However, a double *OsSWEET14*/*OsSWEET13* knockout line in Kitaake is completely resistant to the AXO1947 African strain [7], which represents evidence that those clade III genes are redundant. The expression of other *OsSWEET* genes from clade III is not modified upon BAI3 inoculation in either wild-type or edited lines, suggesting that the loss of *OsSWEET14* induction is not compensated by transcriptional upregulation of redundant homologous genes. Thus, in contrast to Asian *Xoo*, African *Xoo* has the capacity to infect rice in the absence of clade III *SWEET* susceptibility gene induction [35]. These experimental data were unexpected because they are inconsistent with the dramatic phenotype of BAI3-1-1, a BAI3 derivative with a defective *talC* gene that has been previously shown to be unable to cause significant BLB symptoms and efficiently colonize susceptible rice leaves, leading to the conclusion that this effector is a major virulence TALE [26,31]. To reconcile these observations, we previously postulated the existence of an unidentified susceptibility gene targeted by TalC and genetically redundant with *OsSWEET14* [35].

This study was primarily conducted to explore the possible existence of such a redundant TalC target. Revisiting available rice transcriptomics data with an original strategy helped identify a particularly interesting candidate TalC target locus. In addition, we provide evidence that *talC* triggers transcription of two divergent transcripts through the recognition of an EBE that is shown to be necessary for induction at this target locus. However, both gain- and loss-of-function approaches could not establish that this rice locus contributes to BLB susceptibility. Overall, this work identifies a second TalC target and provides the first unambiguous results indicating that a native TALE can trigger bidirectional transcription during infection.

## 2. Results

### 2.1. Reassessing TalC Target Predictions in the Context of a BAI3-Infected Rice Experimental Transcriptome Yields New Candidate TalC Target Loci

Prior searches for TalC targets in rice identified *OsSWEET14* [31]. A subsequent Nipponbare-genome-wide inquiry of African TALE targets combined both in silico target predictions on upstream sequences of annotated genes and lists of differentially expressed genes upon African Xoo infection based on transcriptomic data [25]. In addition to *OsSWEET14*, the latter approach identified LOC_Os03g58790, a putative ATPase, as a candidate TalC target. However, the induction ratio and prediction rank values are moderately supportive for a TALE target (~4× and 264, respectively). Furthermore, the predicted TalC EBE for this locus starts with a T for position 0 of the RVD array, which contrasts with the functional TalC EBE on the *OsSWEET14* promoter, which uncommonly starts with a C, as previously pointed out [31]. Considering both that TALEs may trigger the synthesis of transcripts overlooked in reference annotations and that previous TalC target searches did not incorporate the possibility of “antisense EBEs,” in order to maximize the likelihood of identifying candidate redundant susceptibility genes, we decided to reassess TalC target predictions.

For that, we analyzed a recently documented mRNA-seq dataset [36]. First, an experimental transcriptome (see our dataverse) was inferred with the StringTie software by combining mRNA-seq read alignments to the Nipponbare genome and the reference MSU7 annotation [37]. This experimental transcriptome served to compute read counts at the gene level, which permitted the assessment of differential expression (annotation and expression data are available on our dataverse). From this, we listed 261 loci deemed as differentially induced in comparisons between African-*Xoo*-strain-BAI3-infected leaves and a BAI3 T3SS defective mutant strain (BAI3H) or mock inoculated Nipponbare leaves. To predict potential TalC EBEs, we ran Talvez [38] using the sequences located 500 bp upstream and 200 bp downstream of the start of the experimental BAI3 T3SS upregulated loci. The resulting list of candidate loci with the 10 highest Talvez prediction scores is shown in Table 1. When available, TalC-dependent induction data (derived from 31) were included in this table. Annotated genome browser views of those candidate TalC target loci with read coverage profiles and predicted EBEs are provided in Figure S1.

As expected, the *OsSWEET14* locus (LOC_Os11g31190, also previously designated as Os11N3) carries the top-scoring predicted EBE and exhibits elevated T3SS- and *talC*-dependent induction ratios (Table 1). Apart from this positive control locus, the *INCREASED LEAF INCLINATION 2* (*ILI2*) locus (LOC_Os11g39000) is the only MSU7 reference annotation locus with a similar profile, being notably induced in a *talC*-dependent manner (adjusted *p*-value = 6.29 × 10^−3^; Table 1). In contrast, however, the predicted TalC EBE is positioned in an antisense orientation relative to the ILI2 coding strand.

**Figure 1:**
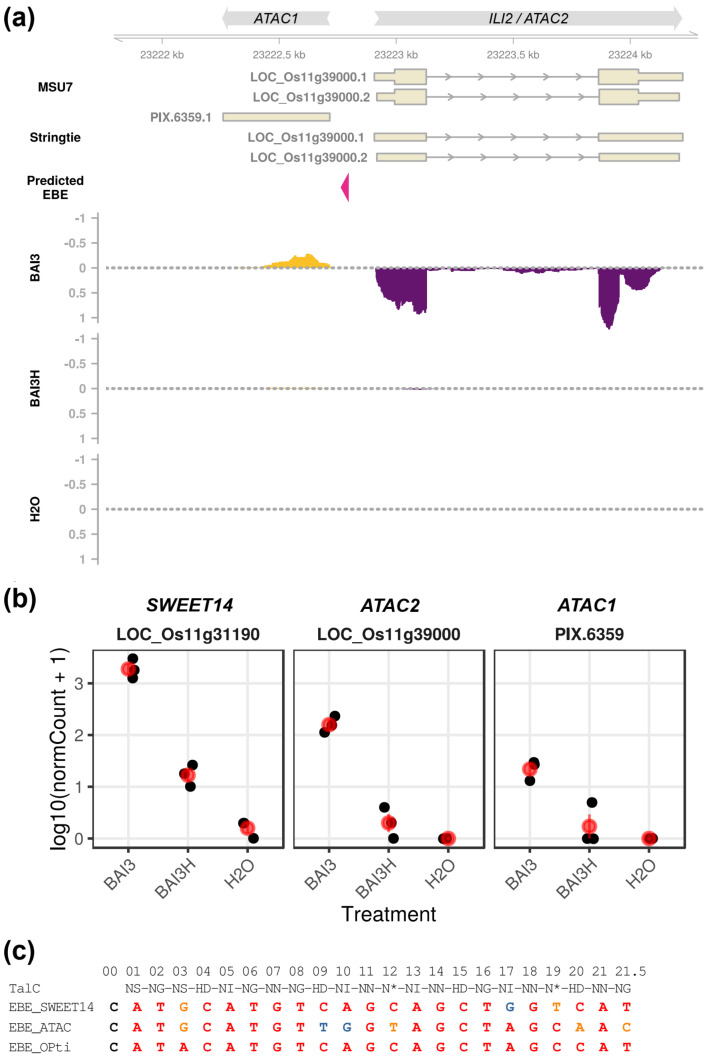
Transcripts at a second candidate target locus are potentially induced bidirectionally by TalC. (**a**) Genome browser snapshot of the ATAC locus with transcript annotation from MSU7, the experimental transcriptome annotation inferred by StringTie, the predicted TalC EBE and read coverage tracks (reads per million) for various experimental treatments in our mRNA-seq data. For the MSU7 annotation, shorter exon boxes correspond to untranslated regions. For coverage tracks, only the reads corresponding to the transcribed strand in paired-end data for a specific treatment were considered. Sequencing libraries were generated with RNA extracted 24 h after infiltration of Nipponbare leaves with the strains BAI3, BAI3H (T3SS-), and H_2_O. Coverage of reads mapping to the top genomic strand is represented with positive values and a golden-colored area, whereas coverage of reads mapping to the opposite strand is represented with negative values and a violet-colored area. (**b**) Expression of TalC target genes in our mRNA-seq data. Black dots correspond to individual replicate normalized count values, while the red dots and line ranges denote the mean and the standard error, respectively. (**c**) Alignment of the TalC RVD sequence and putative DNA target sequences. The numbers on top refer to the classical numbering of repeats in the TALE central repeat domain for TalC. The EBE Opti sequence was generated by concatenating the most probable nucleotide for each RVD in the RVD–nucleotide association matrix used by Talvez. In the alignment, nucleotides are colored according to their match to the corresponding TalC RVD in this matrix: red, best score; orange, intermediate; and blue, worst score.

Interestingly, our analytical approach also uncovered two novel loci, PIX.7679 and PIX.6359, without MSU7 annotation, which appeared to generate mRNA-seq reads only if the bacteria were able to deliver type III effectors (Figure S1 and Figure 1a,b). They were, therefore, noteworthy TalC target candidates even if they could not be assessed immediately with regard to talC-dependent induction because their expression is not measured in microarray data.

### 2.2. ATAC, A Candidate Alternative TalC-Target Locus Is Induced Bidirectionally and Comprises a Long ncRNA and the ILI2 Gene

Upon further scrutiny of the genomic positions of the predicted EBEs in Table 1, we unexpectedly realized that the *ILI2* and PIX.6359 loci actually share the same candidate EBE and are arranged in opposite, head-to-head, orientations on Chromosome 11, with the TalC EBE positioned on the same strand as PIX.6359 (Figure 1a). The interval between the PIX.6359 or *ILI2* start site and the proximal end of this EBE is, respectively, 52 bp or 115 bp. The distance from the PIX.6359 transcript start is consistent with distances usually observed between a TALE EBE and its target transcription start (40–60 bp). This configuration is reminiscent of the bidirectional transcription activation by TALEs previously observed in transient plant expression assays [16]. Because of this rather unique feature and because the observed expression profile of the ILI2 and PIX.6359 transcript was at this stage consistent with their being bidirectional targets of TalC (Figure 1b), we tentatively refer to this locus as ATAC (Alternative TalC Target locus), with PIX.6359 designated as *ATAC1* and *ILI2* as *ATAC2*.

In order to examine if the sequence of the predicted EBE in this region (EBE_TalC_@ATAC) is likely to be targeted by TalC, we aligned this sequence with EBE_TalC_ on the *OsSWEET14* promoter (EBE_TalC_@OsSWEET14) and with an “optimal” EBE_TalC_ sequence based on the Talvez nucleotide–RVD association matrix (Figure 1c). The nine first 5′ nucleotides of EBE_TalC_@ATAC and EBE_TalC_@OsSWEET14 are strictly conserved. This includes the cytosine at position 0, an atypical feature of EBE_TalC_@OsSWEET14. EBE_TalC_@SWEET14 displays only one position conflicting with the RVD–nucleotide code. EBE_TalC_@ATAC contains two such positions but possibly with compensatory better matches at other positions.

The StringTie-inferred ATAC1 transcript is 456 nt long and spans positions 23,222,261 to 23,222,716 on Chromosome 11. It is predicted to be composed of a single exon. The longest predicted ORF is 46aa long (Figure S2), and preliminary searches with blastx on NCBI for proteins similar to putative ATAC1 translation products yielded no meaningful hit outside of *O. sativa* hypothetical proteins. Because this suggested that the ATAC1 transcript does not code for a protein, we used PLncPRO [39] to examine if the ATAC1 transcript is a potential long noncoding RNA (lncRNA). Predicting the ATAC1 transcript status with PlncPRO returned a noncoding probability of 0.965, a value well above 0.8, which is considered the lower bound for high-confidence lncRNAs [39].

The *ATAC2*/*ILI2* locus codes for a putative protein belonging to an atypical group of the bHLH transcription factor family that lacks the basic region of the domain and does not bind DNA. In rice, the ILI clade contains seven homologous genes, including *ILI1*, its founding member. These genes are phylogenetically and functionally related to members of the Arabidopsis PRE family [40]. Characterized proteins of this group positively regulate cell elongation in response to environmental and hormonal cues, but this signaling module has also been implicated in the regulation of Arabidopsis PAMP-triggered immunity (PTI) [41].

**Figure 2:**
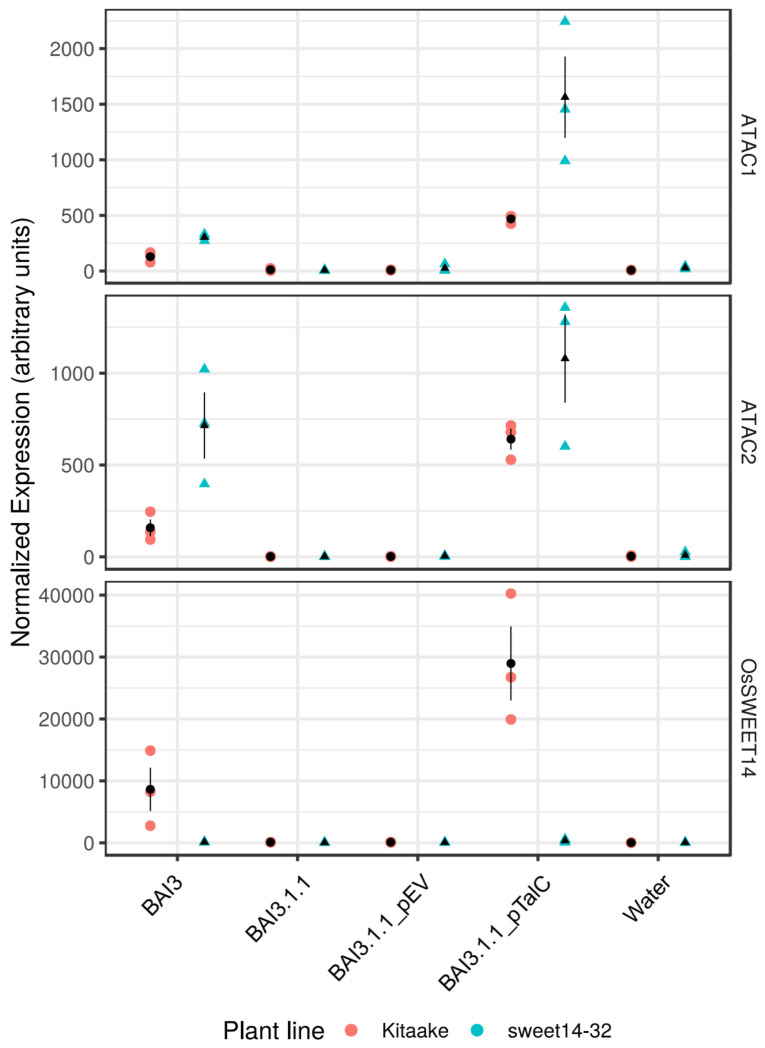
ATAC transcript induction in response to BAI3 is *talC* dependent. Transcript expression of ATAC1, ATAC2, and SWEET14 on the wild-type Kitaake and the edited sweet14–32 lines measured 48 h after inoculation with derivatives of the BAI3 strain or water. The strains are represented on the *x*-axis: BAI3.1.1 is a BAI3 derivative defective for *talC*. “_TalC” or “_EV” suffixes indicate, respectively, that the *talC* strain carried a plasmid expressing TalC or the corresponding empty vector. Expression levels of the different transcripts measured by qRT-PCR are expressed in arbitrary units and calculated by the ∆Ct method. Individual biological replicate values (*n* = 3) are denoted with colored symbols as a function of the inoculated plant line. The black dots and line ranges denote the mean and the standard error, respectively.

Under the LOC_Os11g39000 locus identifier, *ATAC2*/*ILI2* has previously been shown by Yu et al. [31] to be *talC*-regulated based both on microarray and independent RT-PCR data. To ascertain that ATAC1 lncRNA induction is both T3SS and *talC* dependent, we profiled gene expression in response to wild-type BAI3, its *talC*-mutant derivative BAI3-1-1 [31], and complemented strains in leaves of wild-type Kitaake or the EBE_TalC_@OsSWEET14-edited line *sweet14–32* [35]. The qRT-PCR data in Figure 2 indicate that the *OsSWEET14* control gene is induced on wild-type plants but not on the edited line, in response to the BAI3 strain and the BAI3-1-1 mutant complemented with *talC*. ATAC transcripts expression follow a similar pattern except that they also appear to be *talC* induced on the EBE_TalC_@OsSWEET14-edited line. This demonstrates that both *ATAC1* and *ATAC2* are *talC* regulated and that this effect occurs independently of *OsSWEET14* induction.

Altogether, these data support the hypothesis that TalC recognizes a second sequence in the rice genome at the ATAC locus and triggers bidirectional transcription of two opposite units.

### 2.3. Artificial TALEs Targeting the ATAC Locus Do Not Enhance the Virulence of a Xoo Strain Unable to Induce an OsSWEET Gene

To explain why, in contrast to Asian *Xoo*, African *Xoo* virulence is marginally affected on TALE-unresponsive *OsSWEET14* promoter-edited lines, we postulated the existence of another TalC susceptibility gene target, redundant with *OsSWEET14*. Under this model, induction of the redundant factor is expected to compensate for the loss of the susceptibility functions provided by *OsSWEET14* induction. If ATAC acted as such a redundant TalC target, we reasoned that the induction of the ATAC locus should rescue the virulence of an Asian strain that is unable to induce OsSWEET functions.

**Figure 3:**
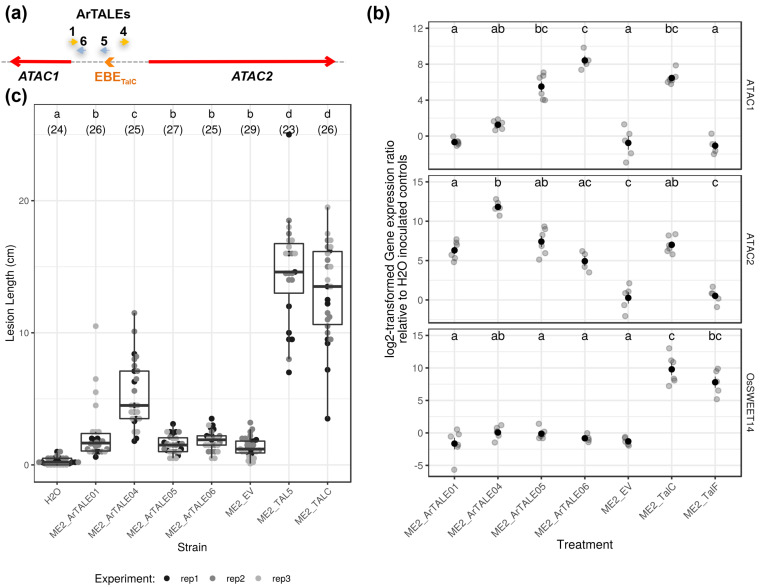
Independent ArTALEs targeting the ATAC loci are functional but do not compensate the loss of clade III OsSWEET induction for the virulence of the Asian PXO99 strain *pthXo1* mutant derivative. (**a**) Diagram depicting the orientations (color and arrowhead) and approximate locations of the different EBEs targeted by ArTALEs relative to the ATAC locus transcripts. Numbers on top of ArTALE EBEs identify individual ArTALEs in panels B and C below. (**b**) TalC target transcript expression ratios in response to ArTALEs designed to target the ATAC locus. qRT-PCR was performed with leaf samples collected 48 h post infiltration of the Kitaake wild-type rice line with derivatives of the ME2 (PXO99 mutated in the major TALE gene *pthXo1*) strain carrying an empty vector (EV) or related plasmids encoding the indicated TALEs. The plotted data (gray dots) derive from two independent experiments (*n* = 4–6). The black dots and line ranges denote the mean and the standard error, respectively. Treatments with the same letter in an individual transcript panel are not significantly different (Dunn’s test for post hoc pairwise multiple comparisons; *p*-value < 0.05). (**c**) Lesion length measured on wild-type rice line Kitaake 14 days post leaf clipping inoculation with the indicated strains. The plotted data derive from three independent experiments (dots with different gray levels). Additional water control leaves did not show BLB symptoms. The boxplots represent the median and the first and third quartiles, whereas the whisker extends from the hinge to the largest or the lowest value but no further than 1.5 times the distance between the first and third quartiles. Treatments with the same letter in an individual transcript panel are not significantly different (Dunn’s test for post hoc pairwise multiple comparisons; *p*-value < 0.05). Numbers in parentheses represent the count of values for the corresponding treatment.

To address this possibility, we assembled a set of four “designer” or “artificial” TALEs (ArTALEs) as before [26]. Their respective EBEs were selected to maximize specificity toward the ATAC loci and as shown in Figure 3a and Figure S3, are located near EBE_TalC_@ATAC on the same strand (ArTALE05 and 06) or the opposite, *ATAC2*, strand (ArTALE01 and 04). These constructs, together with the empty vector or plasmids expressing the control TalF_MAI1_ or TalC_BAI3_ native TALEs, were introduced into the ME2 strain, a non-virulent PXO99A mutant derivative deprived of the PthXo1 TALE [42] that is routinely employed for testing virulence complementation by *OsSWEET* targeting TALEs [8,16]. The resulting strains are described in Table 2. To functionally validate these ArTALEs, the transformed strains were infiltrated into Kitaake leaves and their ability to induce gene expression was monitored with qRT-PCR (Figure 3b). The positive control strain expressing TalC induced *OsSWEET14*, *ATAC1*, and *ATAC2* relative to the empty vector strain, whereas the ME2 strain expressing TalF induced *OsSWEET14* only. None of the ArTALEs were capable of dramatically affecting *OsSWEET14* expression relative to water-infiltrated plants. In contrast, all four of them induced *ATAC2* above empty control levels. The situation regarding *ATAC1* is more contrasted. ArTALE05 and 06 induced this transcript at the same level as TalC, whereas ArTALE01 and, to a lesser extent, ArTALE04 were similar to the empty control strain. Overall, it appeared that all our ArTALEs are able to specifically activate ATAC transcription and that those designed to target an EBE with the same orientation as EBE_TalC_@ATAC, similar to TalC, acted in a bidirectional manner.

Next, to test if ATAC transcript induction could confer enhanced virulence on the ME2 mutant, we screened those strains in leaf clipping virulence assays on Kitaake plants. As shown in Figure 3c, the TalC- and TalF-expressing strains caused disease lesions longer than 10 cm, well above the empty vector control strain and consistent with previous similar experiments [7,8]. However, with the exception of the ME2 derivative expressing ArTALE04, lesion lengths recorded for other ArTALE-expressing strains were not different from those produced with the empty vector control strain.

We, therefore, conclude that while our ArTALEs could simultaneously induce both *ATAC1* and *ATAC2* transcripts or *ATAC2* alone, they did not consistently compensate for the loss of clade III OsSWEET induction and failed to confer increased virulence on the ME2 mutant strain in our conditions.

### 2.4. Multiplex Editing of the OsSWEET14 Promoter and the ATAC Locus Using CRISPR/Cas9

Negative results in these gain-of-function experiments could stem from a number of reasons. If ATAC is indeed redundant with *OsSWEET14*, another prediction of our working model is that TalC-unresponsive plants should display enhanced resistance to African Xoo strains. To investigate the significance of ATAC targeting by TalC with a complementary loss-of-function approach, we therefore applied CRISPR/Cas9 genome editing to inactivate TalC target loci in multiplex. To this end, we employed a previously described system [43] to generate a set of binary constructs with *cas9* and an array of 4 gRNAs (Figure S4) for the genetic transformation of the Nipponbare variety. To abrogate TALE-mediated *OsSWEET14* induction, all our three constructs bore a gRNA targeting EBE_TalF_ and another gRNA targeting EBE_TalC_@OsSWEET14. In order to query different regions of the ATAC loci, they differed at the level of the two remaining gRNAs: a pair of gRNA targeting EBE_TalC_@ATAC or a nearby sequence was included in one construct with the goal of abrogating TalC responsiveness. Additional pairs of gRNAs targeting either *ATAC1* or *ATAC2* transcript sequences for the insertion of deleterious mutations in the underlying locus, including the deletion of intervening sequences, were cloned in the second or third constructs (Figure S4 and Table S1). Finally, two complementary control constructs designed for single locus editing and comprising only the *OsSWEET14* promoter gRNAs or the pair of gRNAs targeting *ATAC2* were also included in transformation experiments.

**Figure 4:**
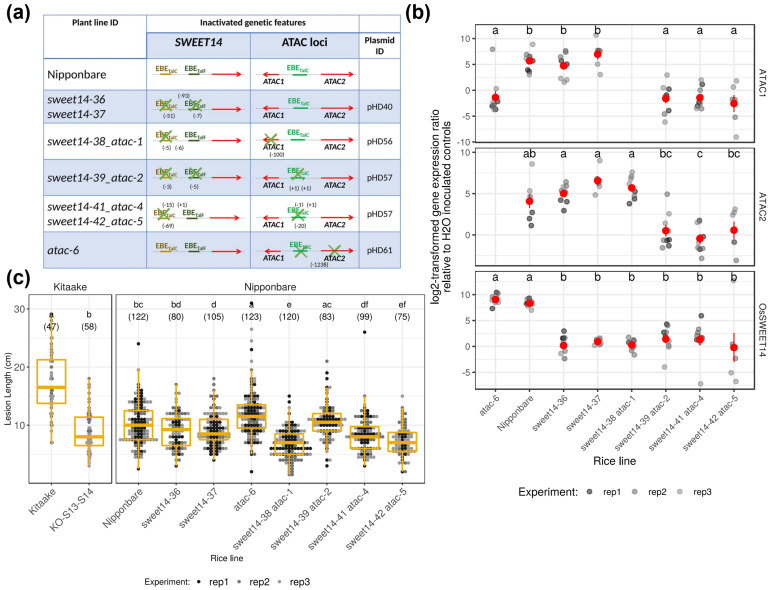
ATAC-locus-edited lines confirm that EBE_TalC_@ATAC is functional but does not support a role as a BLB disease susceptibility locus. (**a**) A schematic diagram describing the nature of the editing event in the modified Nipponbare homozygous lines characterized below. A cross on top of a functional feature indicates that the sequence of the corresponding allele has been altered. Red arrows represent the *OsSWEET14* or *ATAC* genes. Colored segments depict TALE target EBEs. Values in parentheses aligned with a line identifier inform about the extent and approximate location of DNA editions as in Figure S5. Features are not drawn to scale. (**b**) TalC target transcript expression ratios in multiplex edited lines 48 h post infiltration with BAI3 relative to water-infiltrated control leaves. The plotted data (gray dots) derive from three independent experiments (shades of gray; *n* = 6–9). The black dots and line ranges denote the mean and the standard error, respectively. Treatments with the same letter in an individual transcript panel are not significantly different (Dunn’s test for post hoc pairwise multiple comparisons; *p*-value < 0.05). For a couple of genotypes, sequence alterations precluded qRT-PCR on a specific target, and no data are reported (no mean difference letter; see the main text for details). (**c**) Leaf clipping susceptibility assays performed with the edited lines described in panel A challenged with the BAI3 strain. Lesion lengths were measured 14 days post inoculation. Additional water control leaves did not show BLB symptoms. The plotted data derive from three independent experiments (dots with different gray levels). The boxplots represent the median and the first and third quartiles, whereas the whisker extends from the hinge to the largest or the lowest value but no further than 1.5 times the distance between the first and third quartiles. Treatment comparison was performed separately depending on the wild-type rice background, and those with the same letter are not significantly different (Dunn’s test for post hoc pairwise multiple comparisons; *p*-value < 0.05). Numbers in parentheses represent the count of values for the corresponding treatment.

Ultimately, after transformation, genotyping, and propagation of edited plants, we gathered a set of seven homozygous, non-transgenic T3 lines with a range of edition events as described in Figure 4a and Figure S5. The efficiency of EBE_TalF_ editing was too low to select consistently modified lines. However, all sweet14 alleles carry mutations that are likely to prevent TalC recognition. The *atac-1* allele consists in a 100 bp deletion of the *ATAC1* transcript, including a portion of the longest predicted ORF. Furthermore, we obtained several alleles with insertions or deletions upstream of position 8 of EBE_TalC_@ATAC (*attac2*, *atac4*, and *atac5*) (see Figure 4a and Figure S5). We were, however, unable to recover edited lines combining an unresponsive allele at EBE_TalC_@OsSWEET14 and a modified allele of *ATAC2*.

### 2.5. Bidirectional Induction of Transcription at ATAC Is Dependent on the Predicted TalC EBE, but TalC-Unresponsive Plants Remain Essentially Susceptible to an African Xoo Strain

Next, we examined TalC-responsiveness of edited lines using qRT-PCR following leaf infiltration of the BAI3 strain (Figure 4b). As opposed to wild-type Nipponbare or the *atac-6* line, and consistent with our previous results [35], *OsSWEET14* induction was abrogated in all lines carrying a mutation affecting the EBE_TalC_ on its promoter.

Plants containing the *atac-1* allele were not measured for *ATAC1* levels because of the large deletion in the genomic sequence of the transcript. Likewise, the *atac-6* plants were not assayed for *ATAC2* expression because of the deletion of most of the gene sequences, including EBE_TalC_@ATAC (Figure S5). With these potential caveats in mind, plants with the *atac-1* allele were not compromised for *ATAC2* induction that was similar to the wild-type plants. In contrast, in *atac-6* plants, *ATAC1* was not responsive to BAI3 inoculations, possibly because EBE_TalC_@ATAC is missing in those individuals. In agreement with this view, induction was essentially abrogated for both ATAC transcripts in other lines with more subtle modifications at EBE_TalC_@ATAC (*atac2*, *atac4*, and *atac5* alleles). This is indicative that an intact EBE_TalC_@ATAC is necessary for TalC recognition and subsequent divergent transcription activation.

Having determined that our edition events panel encompasses a range of effects relative to the potential function and the regulation of the ATAC loci combined with TalC-unresponsive *OsSWEET14* promoters, we asked if this resulted in altered susceptibility to the *Xoo* strain BAI3. As shown in Figure 4c, while in our leaf clipping assays with BAI3, and as reported previously [7], the double *OsSWEET14*/*OsSWEET13* knockout plants (KO-S13-S14) displayed strongly reduced lesions relative to the wild-type Kitaake controls, the resistance level of our edited plants in the Nipponbare background was not commensurately affected. Even if the lesion lengths for two lines edited in multiplex at both ATAC and *OsSWEET14* loci (sweet14–38 atac-1 and sweet14–42 atac-5) were significantly different than wild-type and single edited lines (*sweet14–36* and *sweet14–37*), this effect was modest and not consistent across modified lines with similar anticipated genetic defects.

Overall, the production and phenotypical analysis of a series of ATAC-edited Nipponbare lines revealed that the putative TalC EBE is required for bidirectional induction of transcription at this locus by BAI3 but simultaneous abrogation of TalC responsiveness at both *OsSWEET14* and ATAC loci did not confer dramatically enhanced resistance to disease caused by the African *Xoo* BAI3 strain.

## 3. Discussion

### 3.1. TalC Functions and the Dependence of Af Xoo on OsSWEET Induction for Infection

In this study, following up on our earlier results, we investigated potential alternative TalC targets in the rice genome and identified a second TalC-responsive locus coined ATAC. Recent data indicate that multi-EBE-edited Kitaake lines with an altered EBE_TalC_@OsSWEET14 are resistant to the ME2 derivative complemented with *talC*, which is otherwise fully virulent on wild-type Kitaake rice [8]. Similarly, *talC* no longer provides increased lesions to ME2 on the *OsSWEET14* knockout line versus wild-type Kitaake [7]. Thus, at least in the ME2 background, *talC* contribution to virulence is abrogated when either host *OsSWEET14* gene activity or transcriptional induction is compromised. This is consistent with the view that *talC* virulence activity is exclusively channeled via an *OsSWEET14* pathway and contradicts our hypothesis that alternative TalC rice targets, including the ATAC locus, could redundantly contribute to susceptibility.

Considering these observations and our inability to demonstrate a contribution of ATAC transcripts using either ArTALEs (Figure 3c) or edited lines (Figure 4c), we provisionally conclude that transcriptional targeting of ATAC is likely fortuitous and does not contribute to the virulence of African strains causing BLB. In this context, the mild ME2-virulence-enhancing activity of ArTALE04 only (Figure 3c) can only be interpreted as an off-target effect of this particular construct.

A growing list of TALEs has been shown to simultaneously target more than one host genomic locus for transcription activation. Most documented examples, such as AvrXa7 on *OsSWEET14* and the transposon Os04g19960 [5], the *X. oryzae* pv. *orzicola* Tal2g on rice *OsSULTR3;6* and Os06g46500 [44], or the *X. citri* subsp. *citri* TALE PthA4 on *CsLOB1* and *CsSWEET1* [45], involve a genuine susceptibility gene and a second host locus that does not measurably affect disease, as is likely the case with TalC. Nonetheless, dual upregulation of independent bona fide susceptibility genes by single TALEs has been demonstrated. For example, the African Xoo TALE TalB targets *OsTFX1* and *OsERF#123*, both of which increase BLB symptoms upon induction [25]. The tomato (*Solanum lycopersicum*) transcription factor genes *bHLH3* (Solyc03g097820) and *bHLH6* (Solyc06g072520) are both regulated by AvrHah1, a TALE from X. gardneri and can individually promote water soaking, a typical disease readout [46].

Incidentally, regardless of whether this impinges on plant–pathogenic bacteria interactions, TALEs appear to repeatedly regulate genes of the large plant basic/helix–loop–helix (bHLH) transcription factor family [47]. In addition to AvrHah1 (mentioned above) and TalC, with the newly discovered *ILI2*/*ATAC2* target reported in this study, an archetypal example of this is AvrBs3 from *X. campestris* pv. *vesicatoria* and its bHLH target *UPA20*, which controls pepper (*Capsicum annuum*) cell hypertrophy [13]. Many strains of the *Xoo*-related *oryzicola* pathovar of *X. oryzae* broadly upregulate the rice Os03g07540 locus, which also encodes a bHLH protein [48]. With the BLS256 strain, this effect is mediated by the Tal3c TALE [44]. Intriguingly, Os03g07540, which corresponds to *ILI3* [40], is therefore a close homolog of ILI2/ATAC2, the alternative TalC target identified here.

### 3.2. Bidirectional Induction of Transcription by TALEs

Arguably, one of the most important outcomes of our work, with relevance to the molecular mechanisms of TALE-mediated transcription activation in plants, is the observation that the TalC bacterial effector triggers divergent bidirectional transcription at ATAC. Both ATAC1 and ATAC2 transcripts are coordinately induced in a TalC-dependent manner (Figure 2), and this requires an intact putative TalC EBE at the ATAC locus (Figure 4).

ATAC1 is a previously undocumented short (456 nt) rice transcript (Figure 1) whose putative translation products are less than 100 amino acids (Figure S2) and that is predicted to be an lncRNA by a classifier trained on a rice-specific dataset. Plant lncRNAs are comparatively less understood than animal lncRNAs, and they comprise a relatively diverse set of molecules having functions in plant development and adaptation to both abiotic and biotic stresses through a variety of mechanisms [49]. Whether *ATAC1* has an intrinsic biological function or is just an accidental by-product of TalC activity is an interesting question that may be illuminated in future work. In line with another study [50], the discovery of the ATAC1 transcript in a reconstructed transcriptome is, nonetheless, a strong hint that the search for TALE target sequences should not solely rely on incomplete reference plant genome annotations and must incorporate experimental RNA-seq information to enable the identification of novel candidates.

Two previous studies have demonstrated that designer TALEs can activate unidirectional transcription of sequences upstream of the EBE on the opposite strand (antisense transcription). This was shown to occur both in transient assays in *Nicotiana benthamiana* and during leaf infection. ArTALEs designed to target an EBE in an antisense orientation relative to *OsSWEET14* [15,16] or *OsSULTR3;6* [16] susceptibility genes were able to complement the virulence of strains deficient for the cognate natural TALE. Wang et al. [16] provided additional insight and showed that both artificial and natural TALEs can activate bidirectional transcription in Agrobacterium-mediated transient expression assays in *N. benthamiana* with a T-DNA carrying a dual reporter construct. The same authors also described two functional EBEs in opposite orientation for the natural *X. oryzae* pv. *orzicola* TALE Tal3c upstream of the Os09g39810 locus, but they detected no sign of bidirectional transcription in mRNA-seq expression data during infection. Here, in contrast, our results represent the first tangible evidence that a native TALE can activate bidirectional transcription when delivered by the T3SS during infection.

ATAC1 is reminiscent of enhancer-associated lncRNAs (eRNAs) [51,52]. While in animals, enhancers and gene promoters frequently undergo divergent RNA-polymerase-II-mediated transcription, in plants, the evidence for bidirectional transcription from promoters of protein-coding genes is relatively scarce and previously defined bidirectional plant promoters [53,54,55,56] may entail a different phenomenon. However, hypomorphic mutations in the Arabidopsis exosome complex or its associated helicase system have recently enabled the detection of about a hundred loci with antisense RNAs upstream of mRNA transcripts and an equivalent number of noncoding regions with signs of bidirectional transcription reminiscent of eRNAs [57]. TALE-driven divergent transcription may be a consequence of an idiosyncratic mode of action of these bacterially encoded transcription factors. This property probably depends on the nature of the additional plant transcription initiation machinery components that are recruited and that are just beginning to be deciphered [58]. The current picture is, however, that TALEs predominantly activate unidirectional transcription downstream of their EBEs; for example, there is no signal of antisense transcription at the *OsSWEET14* (LOC_Os11g31190) promoter (Figure S1). Thus, as suggested before [15,16], other promoter elements (e.g., the TATA-box) but also DNA conformational properties (dictated in part by chromatin modifications) conceivably determine the local mode of transcription activation (i.e., bidirectional, sense or antisense only) by stimulating the release of halted RNA polymerase II complexes or by inhibiting the exosomal processing of nascent transcripts. In this regard, it is probably not fortuitous that our ArTALE05 and 06 with an EBE on the same strand as EBE_TalC_@ATAC trigger divergent transcription whereas ArTALE01 and 04 with an EBE oriented toward *ATAC2* activate unidirectional sense transcription only (Figure 3).

In conclusion, pursuing the hypothesis that TalC targeted a second redundant susceptibility gene in rice, we did identify a second locus but failed to demonstrate its contribution to disease susceptibility. Hence, the basis of the unique ability of African Xoo to persistently colonize rice in the absence of clade III OsSWEET gene induction remains enigmatic and shall be investigated further. In this endeavor, we nonetheless documented the first example of bidirectional transcription activation by a native TALE during infection, which will be a valuable system for a better understanding of the molecular mechanisms underlying TALE activity.

## 4. Materials and Methods

### 4.1. Bacterial Strains, Plant Inoculations, and Growth of Bacteria in Planta

The wild-type rice genotypes Kitaake and Nipponbare varieties used in this study were originally obtained from the International Rice Research Institute and the IRGSP consortium [37], respectively. The *OsSWEET14*/*OsSWEET13* double-knockout edited line corresponds to the *sweet13;14* +1bp/+1bp line from Eom et al. [7]. All rice plants were grown under cycles of 12 h of light at 28 °C and 80% relative humidity (RH) and 12 h of darkness at 25 °C and 70% RH in the greenhouse.

The plasmids and *Xoo* bacterial strains used in this study are described in Tables S1 and S2, respectively. Bacterial strains stored in 15% glycerol at −80 °C were cultivated on PSA (10 g/L peptone, 10 g/L sucrose, 1 g/L glutamic acid, and 16 g/L Bacto Agar). The strains containing plasmids were grown on PSA supplemented as needed with kanamycin, gentamicin, or rifampicin at concentrations of 50 μg/mL, 20 μg/mL, and 50 μg/mL, respectively. The cultures were incubated at 28 °C for 48 h.

Inoculations were conducted as before [31]. Briefly, bacteria grown on plates for 48 h were resuspended in sterile water at an OD600 = 0.2 for leaf clipping assay inoculum preparation or OD600 = 0.5 for leaf infiltration. Leaves of 6-week-old plants were cut with scissors previously dipped in bacterial suspensions, whereas leaves of 4-week-old plants were pressure infiltrated with a 1 mL syringe. Lesion lengths were measured 14 days post inoculation.

### 4.2. RNA Isolation and qRT-PCR

The leaves of Kitaake, Nipponbare, or edited rice line plants were infiltrated with bacterial suspensions or water, and three independent replicate samples were processed at 48 h after inoculation. Total RNA was extracted using TRI reagent (EUROMEDEX, Souffelweyersheim, France) and further purified using the RNA Clean-Up & Concentration kit (Zymo Research, Irvine, CA, USA) according to the manufacturer’s instructions. A total of 1 μg of RNA per sample was treated with the TURBO DNA-free kit (Thermo Fisher Scientific, Waltham, MA, USA) and reverse-transcribed into cDNA using SuperScriptIII (Thermo Fisher Scientific, Waltham, MA, USA) with oligo-dT primers. PCR reactions were performed with SYBR-based Mesa Blue qPCR Mastermix (EUROGENTEC, Liège, Belgium).

For the OsSWEET14 gene, qRT-PCR oligos were reported before [35]. For the ATAC locus, a primer pair specific to the ATAC1 transcript (GAGGTGGAGCTGGAGCTATG and TACAACGGGCTACAACCACA) and a primer pair specific to the ATAC2 transcript (CGTCCGATCTCGCCATCAAC and GAGAACATTTTAGGCCGACAGCA) were used. The elongation gene factor-1 alpha (GenBank: GQ848072.1) was used for normalization of the expression values of complementary DNA in each sample as before [35]. Normalized expression values were calculated with the 2^−ΔCt^ methods, whereas expression ratios were calculated with the 2^−ΔΔCt^ method.

### 4.3. Plasmid Vector Construction

The design and construction of artificial TAL effector (ArTALEs) expression vectors involved the use of the method of Streubel et al. [26].

For rice genome editing, first, the pHD46 vector was generated by PCR amplifying the dual gRNA cassette from pENTR4:gRNA4 [43] with a pair of primers wherein the forward primer (ACTTAAGCTTGAATTCagaacgaactaagccggaca) comprises the HindIII and EcoRI restriction sites and the reverse primer (ACCTTTCTAGAgcccttcgaagggacaaaaa) comprises an XbaI site. The amplified sgRNA cassette was digested and gel-purified using the DNA Gel Recovery Kit (Zymo Research, Irvine, CA, USA). This new cassette was cloned into the original pENTR4:gRNA4 vector digested at the EcoRI and XbaI restriction sites that are located in between the attL sequences.

The E-CRISPR software [59] was used to design synthetic guide RNAs (sgRNAs). The sgRNA sequences are indicated in Figure S4. When the designed gRNA sequence 5′ nucleotide was not a guanine (G), a starting G was appended to this sequence to promote U6 promoter transcription initiation at this site. Complementary oligos spanning sgRNA sequences with appropriate 4 bp overhangs were thermally annealed to each other, diluted (1:200) with distilled water, and cloned into pENTR4:gRNA4 or pHD46 vectors using the procedure described by Zhang et al. [43].

For the assembly of an array of four gRNA expression units into a Gateway entry vector, pHD46-derived EcoRI-XbaI cassettes with ATAC locus targeting gRNAs were cloned into pHD23, a pENTR4:gRNA4-based vector bearing the OsSWEET14 promoter targeting gRNAs, digested with the same enzymes. This array was subsequently transferred into a modified pH-Ubi-cas9-7 [60] vector documented by Fayos et al. [61] in a Gateway LR reaction (Thermo Fisher Scientific, Waltham, MA, USA). The gRNA arrays of the resulting binary vectors (see Table S1) were confirmed again by sequencing before Agrobacterium transformation was continued.

### 4.4. Genetic Transformation of Rice and Modified Allele Genotyping

For rice genetic transformation, *Agrobacterium tumefaciens* EHA105 was transformed with the set of binary vectors by electroporation. The embryo calli derived from Nipponbare were used and co-cultured with bacteria as described [35,62]. T0 plants were screened to characterize multiplex editing events. The MATAB protocol [63] was used to extract DNA from leaf samples. The amplification products of the OsSWEET14 promoter were obtained as described before [35]. The ATAC locus was PCR amplified with specific forward (GACTGTAGCTTTGCCTAGCT) and reverse (CTACAGATGAAGAAGCTAGAG) primers. The resulting amplicons were Sanger sequenced and analyzed with DSDecode software [64] to identify mutations within the *OsSWEET14* promoter. For simultaneous detection of mutation of the *OsSWEET14* promoter and ATAC locus, the PCR products were cloned into the pGMT-T vector according to the instructions of the manufacturer (Promega, Madison, WI, USA). The clones were sequenced and analyzed with DSDecode software to decode the mutations.

### 4.5. Computational Methods for TalC Candidate Target Identification

The mRNA-seq data used in this study for experimental transcriptome inference and differential expression analysis are available in SRA (NCBI BioProject accession: PRJNA679478) and have been described before [36].

Paired-end reads were trimmed with AdapterRemoval version 2.2.2 [65] using the following relevant parameters: “--combined-output --gzip --trimns --trimqualities --mm 5 --minadapteroverlap 3 --minlength 25”. Trimmed reads were subsequently mapped onto the nuclear rice genome. Rice Nipponbare IRGSP-1.0 pseudomolecule sequences and MSU7 gene annotation data [37] were obtained from MSU (http://rice.uga.edu/ (accessed on 10 March 2022)). For mapping, HISAT2 (v.2.1.0) was run with the following parameters: “-q --new-summary –dta”. Experimental transcriptome reconstruction essentially followed the recommended procedure [66]. Transcripts were first inferred based on mRNA-seq mappings and the MSU7 reference annotation on an individual sample basis with StringTie (v.1.3.4d) and the following parameters: “-l GXY-1 --fr -m 100 -t -f 0.1 -c 5 -j 2”. Individual gtf files were subsequently merged with the reference annotation in a single step with the StringTie --merge mode and default parameter values.

Gene-level and strand-aware mated read pair counts were obtained with R (v.3.5.1) using the summarizeOverlaps function from the GenomicAlignments (v.1.18.1) Bioconductor package [67], discarding reads overlapping multiple features (“Union” mode and “inter.feature” true). StringTie gene loci that accumulated less than 5 total reads across all libraries in the dataset were excluded. Likewise, loci that overlapped rice loci with feature types tRNA, rRNA, or pseudogenic_tRNA in the gff annotation file for NCBI RefSeq assembly accession GCF_001433935.1 were excluded from differential expression (DE) analysis.

Differential expression was estimated with the DESeq2 (v.1.22.2) R Bioconductor package [68]. For normalization, size factors were estimated using the iterate method and the shorth function to compute a location for a sample. Dispersions were fitted using the parametric method. Two-tailed Wald tests were used for DE tests where the alternative hypothesis was that the absolute value of the log2 transform of the fold change ratio is greater than 0.5. Adjusted *p*-values were computed with the Benjamini & Hochberg method and considered significant when equal to or below 0.1. Outlier filtering based on Cook’s distance was implemented.

To generate a list of upstream sequences for EBE prediction, the DESeq2 gene list was arbitrarily further filtered to retain only the loci with a log2(FC) value ≥ 2 and an adjusted *p*-value ≤ 0.01 in both BAI3 vs. BAI3H and BAI3 vs. H_2_O comparisons. EBE predictions for the TalC RVD sequence on this set of upstream sequences (−500 bp to +200 bp relative to annotated gene start) used Talvez (v.3.2) with parameters “-t 0 -l 19” and default RVD–nucleotide association matrixes [38].

The microarray expression data used to define TalC-induced genes were published earlier [31] and analyzed as previously described [38]. Only probesets with a log2 transform of the fold change ratio ≥ 1 in the XooBAI3-XooBAI3_Dtalc comparison were considered.

The PlncPRO [39] model for *O. sativa* was built using data and instructions from https://github.com/urmi-21/PlncPRO/blob/master/examples/Build%20and%20test%20monocot%20model.ipynb (accessed on 23 July 2021). The plncpro predict (“-v -r”) command was run using the StringTie-predicted ATAC1 transcript sequence.

Data plots were generated in R (v. 3.6.3) with the ggplot2 (v.3.3.2) or Gviz (v.1.30.3) packages, and statistical tests were conducted with functions from the rstatix (v.0.6.0) package.

## Figures and Tables

**Table 1:** Candidate TalC targets in the Nipponbare genome.

EBE Position on IRGSP-1.0				EBE on Subject [−500, +200]			Talvez Score	Candidate Target Gene Annotation				mRNA-Seq Diff. Exp. BAI3 vs. BAI3H			Micro-Array Diff. Exp BAI3 vs. BAI3-1-1 *	
Seq name	start	end	strand	start	end	pol ^‡^		id	ref_gene_id	name_Gramene	description_Gramene	log2FC	pvalue	padj	log2FC	padj
Chr11	18,174,547	18,174,569	-	410	432	S	14.45	PIX.6085	LOC_Os11g31190	11N3	Sugar transporter, TAL effector-mediated susceptibility to bacterial pathogen (Os11t0508600-01)	6.869	2.55 × 10^−45^	8.05 × 10^−42^	5.97	1.03 × 10^−1^
Chr12	17,463,497	17,463,519	+	577	599	S	13.86	PIX.7679	NA ^§^	NA	NA	5.446	1.13 × 10^−12^	4.82 × 10^−10^	NA	NA
Chr5	29,688,055	29,688,077	-	106	128	AS	12.36	PIX.19089	LOC_Os05g51750	NA	Peptidase A1 domain containing protein (Os05t0596000-01)	7.077	5.47 × 10^−37^	9.09 × 10^−34^	NA	NA
Chr11	23,222,769	23,222,791	-	426	448	S	11.27	PIX.6359	NA	NA	NA	4.623	2.81 × 10^−3^	2.19 × 10^−1^	NA	NA
Chr11	23,222,769	23,222,791	-	363	385	AS	11.27	PIX.6360	LOC_Os11g39000	INCREASED LEAF INCLINATION 2	Basic helix–loop–helix dimerization region bHLH domain containing protein (Os11t0603000-01)	6.998	1.09 × 10^−13^	5.08 × 10^−11^	4.26	6.29 × 10^−3^
Chr7	21,798,885	21,798,907	-	496	518	S	10.83	PIX.22524	LOC_Os07g36430	NA	Conserved hypothetical protein (Os07t0549100-01)	4.66	3.82 × 10^−3^	2.83 × 10^−1^	NA	NA
Chr11	28,760,402	28,760,424	+	84	106	AS	10.74	PIX.6663	LOC_Os11g47600	NA	Similar to Class III chitinase homologue (OsChib3H-h) (Fragment) (Os11t0702100-01)	2.432	7.94 × 10^−7^	1.77 × 10^−4^	NA	NA
Chr10	18,168,970	18,168,992	+	369	391	S	10.46	LOC_Os10g34040	LOC_Os10g34040	FLOT1	Similar to Flotillin-like protein 1 (Os10t0481500-00)	4.599	1.68 × 10^−3^	1.39 × 10^−1^	NA	NA
Chr4	25,888,105	25,888,127	+	659	681	AS	10.39	PIX.16030	LOC_Os04g43730	WALL-ASSOCIATED KINASE GENE 51	Similar to OSIGBa0145M07.8 protein (Os04t0517700-01)	7.301	2.80 × 10^−3^	2.19 × 10^−1^	NA	NA
Chr4	15,500,717	15,500,739	-	360	382	S	10.22	PIX.15197	LOC_Os04g26550	NA	Conserved hypothetical protein (Os04t0332700-01)	3.877	5.94 × 10^−13^	2.61 × 10^−10^	NA	NA

* Dataset GSE19844; ^§^ Not Available; ^‡^ Polarity; S, sense; AS, antisense.

**Table 2:** List of *X. oryzae* strains used in this study.

Designation	Strain ID	Description	References
BAI3	CIX151	A rifampicin-resistant and fully virulent derivative of the BAI3 wild-type *Xoo* strain from Burkina Faso	[33]
BAI3H	CIX3878	CIX151 deleted for a portion of the *hrcC* gene	[31]
BAI3.1.1	CIX531	Also referred to as BAI3^R^ Δ*talC*; corresponds to CIX151 with a suicide plasmid insertion in *talC*	[31]
BAI3.1.1_pEV	CIX2533	CIX531 carrying the empty pSKX1 plasmid	[26]
BAI3.1.1_pTalC	CIX2529	CIX531 carrying the pSKX1plasmid containing the *talC* gene from strain BAI3	[26]
ME2	CIX4497	PXO99^A^ derivative with a knockout mutation in *pthXo1*	[42]
ME2_EV	CIX4498	CIX4497 carrying the empty pSKX1 plasmid	This study
ME2_pTALC	CIX4499	CIX4497 carrying the pSKX1plasmid containing the *talC* gene from strain BAI3	This study
ME2_pTALF	CIX4500	CIX4497 carrying the pSKX1plasmid containing the *talF* gene from strain MAI1	This study
ME2_pArTAL01	CIX4501	CIX4497 carrying the pHD62 plasmid expressing ArtTale1	This study
ME2_pArTAL04	CIX4502	CIX4497 carrying the pHD65 plasmid expressing ArtTale4	This study
ME2_pArTAL05	CIX4503	CIX4497 carrying the pHD66 plasmid expressing ArtTale5	This study
ME2_pArTAL06	CIX4504	CIX4497 carrying the pHD67 plasmid expressing ArtTale6	This study

## Data Availability

Data are contained within the article and supplementary material on our dataverse at https://doi.org/10.23708/XBTRUV, last accessed on 23 July 2021.

## Supplementary Materials

The following supporting information can be downloaded at https://www.mdpi.com/article/10.3390/ijms23105559/s1.
