# Supplementary material for: The Rice ILI2 Locus Is a Bidirectional Target of the African Xanthomonas oryzae pv. oryzae Major Transcription Activator-like Effector TalC but Does Not Contribute to Disease Susceptibility
Source: Int J Mol Sci. 2022 May 16;23(10):5559. doi: 10.3390/ijms23105559 (PMC9142087; doi:10.3390/ijms23105559)
Supplement: Supplementary file 1 [file ijms-23-05559-s001.zip › Fig_S3.pdf]

A

ArTALE Name	RVD sequence	Target DNA sequence	Downstream Target Transcript
Art-TALE1	HD-HD-NG-NK-NI-HD-NG-NI-NI-HD-NI-HD-NG-NG-NG-NI-NI-HD	T CCTGACTAACACTTTAAC	ATAC2 / ILI2
Art-TALE4	NN-HD-NI-NG-NN-NG-HD-NG-HD-NG-NI-NG-HD-NI-HD-HD-NN-NG	T GCATGTCTCTATCACCGT	ATAC2 / ILI2
Art-TALE5	NI-NN-HD-NG-NI-NH-HD-NI-NI-HD-NI-NI-NG-NI-NI-NI-NG-NI	T AGCTAGCAACAATAAATA	ATAC1
Art-TALE6	HD-NG-HD-NI-HD-NI-HD-NN-NN-NG-NG-NI-NI-NI-NN-NG-NN-NG	T CTCACACGGTTAAAGTGT	ATAC1

B

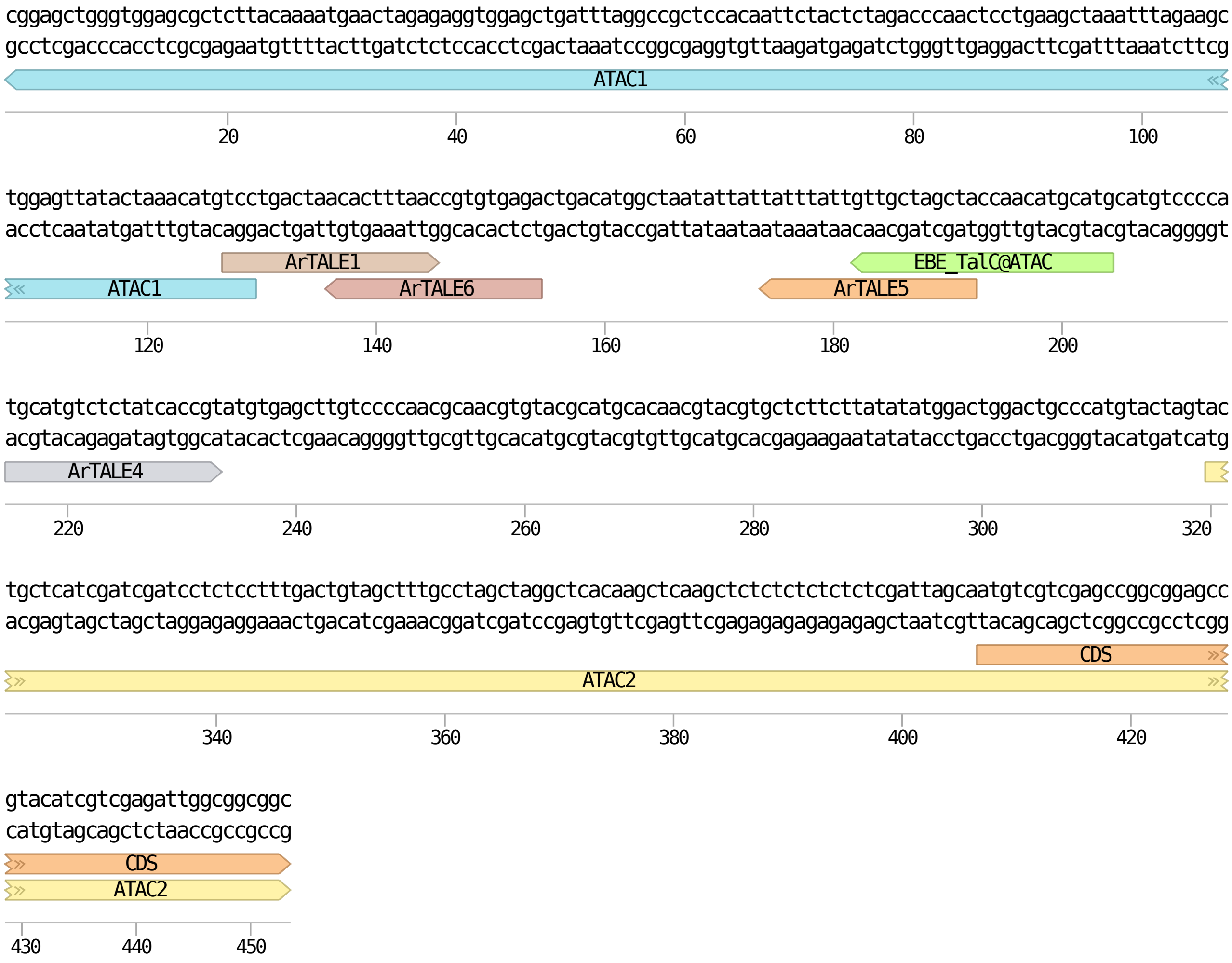

Figure S3
