# Supplementary material for: The Rice ILI2 Locus Is a Bidirectional Target of the African Xanthomonas oryzae pv. oryzae Major Transcription Activator-like Effector TalC but Does Not Contribute to Disease Susceptibility
Source: Int J Mol Sci. 2022 May 16;23(10):5559. doi: 10.3390/ijms23105559 (PMC9142087; doi:10.3390/ijms23105559)
Supplement: Supplementary file 1 [file ijms-23-05559-s001.zip › Fig_S5.pdf]

OsSWEET14 - LOC_Os11g31190

```
                              TalC_EBE                  AvrXa7_EBE               Tal5_EBE
SWEET14p             gggcatgcatgtcagcagctggtcat//atataaaccccctccaaccaggt-gctaagctcatcaagccttcaagcaaagcaaa WT
sweet14-36           gggcatgcatgtc-------------//---------------------------------------------agcaaagcaaa -93
sweet14-37           --------------------------//atataaaccccctccaaccaggt--------tcatcaagccttcaagcaaagcaaa -51-7
sweet14-38 atac-1    gggcatgcatgtcag-----ggtcat//atataaaccccctccaacca-------aagctcatcaagccttcaagcaaagcaaa -5-6
sweet14-39 atac-2    gggcatgcatgtcagc---tggtcat//atataaaccccctccaaccaggt-gc-----tcatcaagccttcaagcaaagcaaa -3-5
sweet14-41 atac-4    g---------------agctggtcat//atataaaccccctccaaccaggtTgctaagctcatcaagccttcaagcaaagcaaa -15+1
sweet14-42 atac-5    gggcatgcatgtcagc----------//------------------------gctaagctcatcaagccttcaagcaaagcaaa -69
atac6                gggcatgcatgtcagcagctggtcat//atataaaccccctccaaccaggt-gctaagctcatcaagccttcaagcaaagcaaa WT
```

ATAC1

```
ATAC1                tacaaccacaagatatctttatccttaaactatacta // tttcagccccggagctgggtggagcgctcttacaaaatgaacta WT
sweet14-36           tacaaccacaagatatctttatccttaaactatacta // tttcagccccggagctgggtggagcgctcttacaaaatgaacta WT
sweet14-37           tacaaccacaagatatctttatccttaaactatacta // tttcagccccggagctgggtggagcgctcttacaaaatgaacta WT
sweet14-38 atac-1    tacaaccacaa--------------------------//  ------------agctgggtggagcgctcttacaaaatgaacta -100
sweet14-39 atac-2    tacaaccacaagatatctttatccttaaactatacta // tttcagccccggagctgggtggagcgctcttacaaaatgaacta WT
sweet14-41 atac-4    tacaaccacaagatatctttatccttaaactatacta // tttcagccccggagctgggtggagcgctcttacaaaatgaacta WT
sweet14-42 atac-5    tacaaccacaagatatctttatccttaaactatacta // tttcagccccggagctgggtggagcgctcttacaaaatgaacta WT
atac6                tacaaccacaagatatctttatccttaaactatacta // tttcagccccggagctgggtggagcgctcttacaaaatgaacta WT
```

ATAC2 – ILI2 - LOC_Os11g39000

```
                           TalC EBE
ATAC2 promoter       tattgttgctagctaccaaca-tgcatgcatgtccccatg-catgtctctatcaccgtatgtgagcttgtccccaacgcaacgt  WT
sweet14-36           tattgttgctagctaccaaca-tgcatgcatgtccccatg-catgtctctatcaccgtatgtgagcttgtccccaacgcaacgt  WT
sweet14-37           tattgttgctagctaccaaca-tgcatgcatgtccccatg-catgtctctatcaccgtatgtgagcttgtccccaacgcaacgt  WT
sweet14-38 atac-1    tattgttgctagctaccaaca-tgcatgcatgtccccatg-catgtctctatcaccgtatgtgagcttgtccccaacgcaacgt  WT
sweet14-39 atac-2    tattgttgctagctaccaacaGtgcatgcatgtccccatgCcatgtctctatcaccgtatgtgagcttgtccccaacgcaacgt +1+1
sweet14-41 atac-4    tattgttgctagctaccaaca--gcatgcatgtccccatgTcatgtctctatcaccgtatgtgagcttgtccccaacgcaacgt -1+1
sweet14-42 atac-5    tattgttgctagctaccaa----------------------catgtctctatcaccgtatgtgagcttgtccccaacgcaacgt -20
atac6                ta---------------------------------------------------------------------------------- -1238

ATAC2 CDS            atgtcgtcgagccggcggagccgtacatcgtcgagattgg // cggcgcagatgtgcccgacctcatccgtagcctactcat  WT
sweet14-36           atgtcgtcgagccggcggagccgtacatcgtcgagattgg // cggcgcagatgtgcccgacctcatccgtagcctactcat  WT
sweet14-37           atgtcgtcgagccggcggagccgtacatcgtcgagattgg // cggcgcagatgtgcccgacctcatccgtagcctactcat  WT
sweet14-38 atac-1    atgtcgtcgagccggcggagccgtacatcgtcgagattgg // cggcgcagatgtgcccgacctcatccgtagcctactcat  WT
sweet14-39 atac-2    atgtcgtcgagccggcggagccgtacatcgtcgagattgg // cggcgcagatgtgcccgacctcatccgtagcctactcat  WT
sweet14-41 atac-4    atgtcgtcgagccggcggagccgtacatcgtcgagattgg // cggcgcagatgtgcccgacctcatccgtagcctactcat  WT
sweet14-42 atac-5    atgtcgtcgagccggcggagccgtacatcgtcgagattgg // cggcgcagatgtgcccgacctcatccgtagcctactcat  WT
atac6                ---------------------------------------- // ------------gcccgacctcatccgtagcctactcat -1238
```

Figure S5
