# Supplementary material for: The Rice ILI2 Locus Is a Bidirectional Target of the African Xanthomonas oryzae pv. oryzae Major Transcription Activator-like Effector TalC but Does Not Contribute to Disease Susceptibility
Source: Int J Mol Sci. 2022 May 16;23(10):5559. doi: 10.3390/ijms23105559 (PMC9142087; doi:10.3390/ijms23105559)
Supplement: Supplementary file 1 [file ijms-23-05559-s001.zip › Fig_Sup_Legends.pdf]

SUPPLEMENTARY FIGURES LEGENDS

Figure S1: Genome browser snapshots of top candidate TalC target genes. MSU7 and experimental (Stringtie) transcripts annotations are represented with connected boxes representing exons. Shorter exon boxes correspond to untranslated regions. Pink arrowheads depict EBEs location and orientation (5' to 3'). For coverage tracks, only reads corresponding to the transcript strand in directional RNA-seq libraries were considered. Sequencing libraries were generated with RNA extracted 24h after infiltration of Nipponbare leaves with the strains BAI3, BAI3H (T3SS-) and H_2O. Coverage of reads mapping to the top genomic strand is represented with positive values and a golden colored area whereas coverage of reads mapping to the opposite strand is represented with negative values and a violet colored area.

Figure S2: Predicted Open-Reading-Frames on the ATAC1 transcript.
The sequence of the transcript strand is displayed with potential codons on the three frames. Predicted ORF (minimum 20 amino acids and allowing alternative start codons in the standard genetic code) are highlighted in golden colored boxes.

Figure S3: Detailed features of the ArTALEs designed to target the ATAC locus.
A. Main features of the ArTALEs constructed for this study.
B. Location of the target EBEs of the ArTALEs in the context of other functional elements on the ATAC loci sequence. Note that for ATAC1 and ATAC2 only incomplete, proximal sequences are shown.

Figure S4: Location of the gRNAs cleavage sites on the sequences targeted for genome editing of the *OsSWEET14* and *ATAC* loci.

Figure S5: Sequences of the edited alleles at the OsSWEET14 and ATAC loci.
A relevant segment of the wild-type sequence is displayed in the first row of the multiple alignment for individual edited loci. The sequences of the edited rice (cv. Kitaake) lines characterized in this study are displayed underneath and are labeled on the left side with the same line identifiers as in Figure 4. A short qualifier for the modification introduced during the editing process is specified on the right. The portions of sequences between two consecutive forward slashes are hidden from the alignment. Deletions and insertions are represented by dashes and bold, capital letters with a yellow background, respectively. Colored arrows on top of wild-type sequences and accordingly colored sequences in the alignments extend over the documented EBEs for Xoo TALEs in these regions.
