# Supplementary material for: The Rice ILI2 Locus Is a Bidirectional Target of the African Xanthomonas oryzae pv. oryzae Major Transcription Activator-like Effector TalC but Does Not Contribute to Disease Susceptibility
Source: Int J Mol Sci. 2022 May 16;23(10):5559. doi: 10.3390/ijms23105559 (PMC9142087; doi:10.3390/ijms23105559)
Supplement: Supplementary file 1 [file ijms-23-05559-s001.zip › Supp_Table01_plasmids.pdf]

Table S1: List of plasmids used in this study.

Plasmid ID	Description	References
pHD40	A modified pH-Ubi-cas9-7 with a castor bean catalase intron in the hpt gene expressing gRNAs 01 and 02 of Figure S4.	This study
pHD46	A pENTR4:gRNA4-based Gateway entry vector for cloning and expression of two gRNA units with flanking EcoRI-XbaI restriction sites.	This study
pHD56	As pHD40 but for expression of gRNAs 01, 02, 03 and 04 of Figure S4.	This study
pHD57	As pHD40 but for expression of gRNAs 01, 02, 05 and 06 of Figure S4.	This study
pHD61	As pHD40 but for expression of gRNAs 07 and 08 of Figure S4.	This study
pSKX1	A Golden Gate-compatible pBBR1MCS-5 derivative with a lac promoter for insert transcription and the FLAG epitope coding sequence for C-terminal fusions.	[26]
pTalC	pSKX1 plasmid with the *talC* gene from the burkinabe strain BAI3	[26, 31]
pTalF	pSKX1 plasmid with the *talF* gene from the malian strain MAI1	[26, 31]
pHD62	SKX1 plasmid with ArtTale1 (Figure S3) coding sequences	This study
pHD65	SKX1 plasmid with ArtTale4 (Figure S3) coding sequences	This study
pHD66	SKX1 plasmid with ArtTale5 (Figure S3) coding sequences	This study
pHD67	SKX1 plasmid with ArtTale6 (Figure S3) coding sequences	This study
