# Supplementary material for: The Rice ILI2 Locus Is a Bidirectional Target of the African Xanthomonas oryzae pv. oryzae Major Transcription Activator-like Effector TalC but Does Not Contribute to Disease Susceptibility
Source: Int J Mol Sci. 2022 May 16;23(10):5559. doi: 10.3390/ijms23105559 (PMC9142087; doi:10.3390/ijms23105559)
Supplement: Supplementary file 1 [file ijms-23-05559-s001.zip › Supp_Table02_Strains.pdf]

Table S2: List of *Xanthomonas oryzae* strains used in this study.

Designation	Strain ID	Description	References
BAI3	CIX151	A rifampicin resistant and fully virulent derivative of the BAI3 wild type Xoo strain from Burkina Faso	[33]
BAI3H	CIX3878	CIX151 deleted for a portion of the *hrcC* gene	[31]
BAI3.1.1	CIX531	This strain is also referred to as $BAI3^{R}$ Δ*talC*. Corresponds to CIX151 with a suicide plasmid insertion in *talC* (3,7kb).	[31]
BAI3.1.1_pEV	CIX2533	CIX531 carrying the empty pSKX1 plasmid	[26]
BAI3.1.1_pTalC	CIX2529	CIX531 carrying pSKX1plasmid containing the *talC* gene from strain BAI3	[26]
ME2	CIX4497	$PXO99^{A}$ derivative with a knockout mutation in *pthXo1*	[42]
ME2_EV	CIX4498	CIX4497 carrying the empty pSKX1 plasmid	This study
ME2_pTALC	CIX4499	CIX4497 carrying pSKX1plasmid containing the *talC* gene from strain BAI3	This study
ME2_pTALF	CIX4500	CIX4497 carrying pSKX1plasmid containing the *talF* gene from strain MAI1	This study
ME2_pArTAL01	CIX4501	CIX4497 carrying the pHD62 plasmid	This study
ME2_pArTAL04	CIX4502	CIX4497 carrying the pHD65 plasmid	This study
ME2_pArTAL05	CIX4503	CIX4497 carrying the pHD66 plasmid	This study
ME2_pArTAL06	CIX4504	CIX4497 carrying the pHD67 plasmid	This study
