# Supplementary figures and images for: The Rice ILI2 Locus Is a Bidirectional Target of the African Xanthomonas oryzae pv. oryzae Major Transcription Activator-like Effector TalC but Does Not Contribute to Disease Susceptibility
Source: Int J Mol Sci. 2022 May 16;23(10):5559. doi: 10.3390/ijms23105559 (PMC9142087; doi:10.3390/ijms23105559)

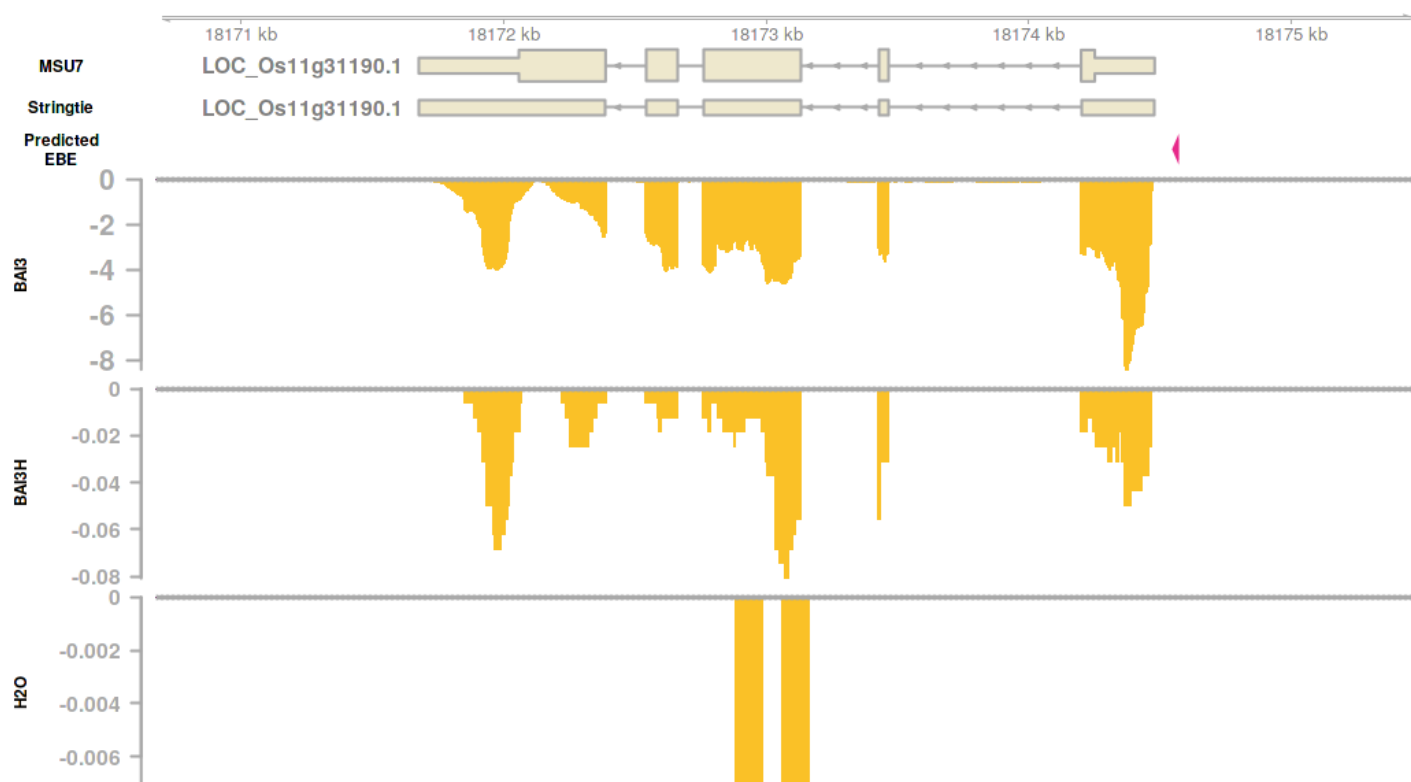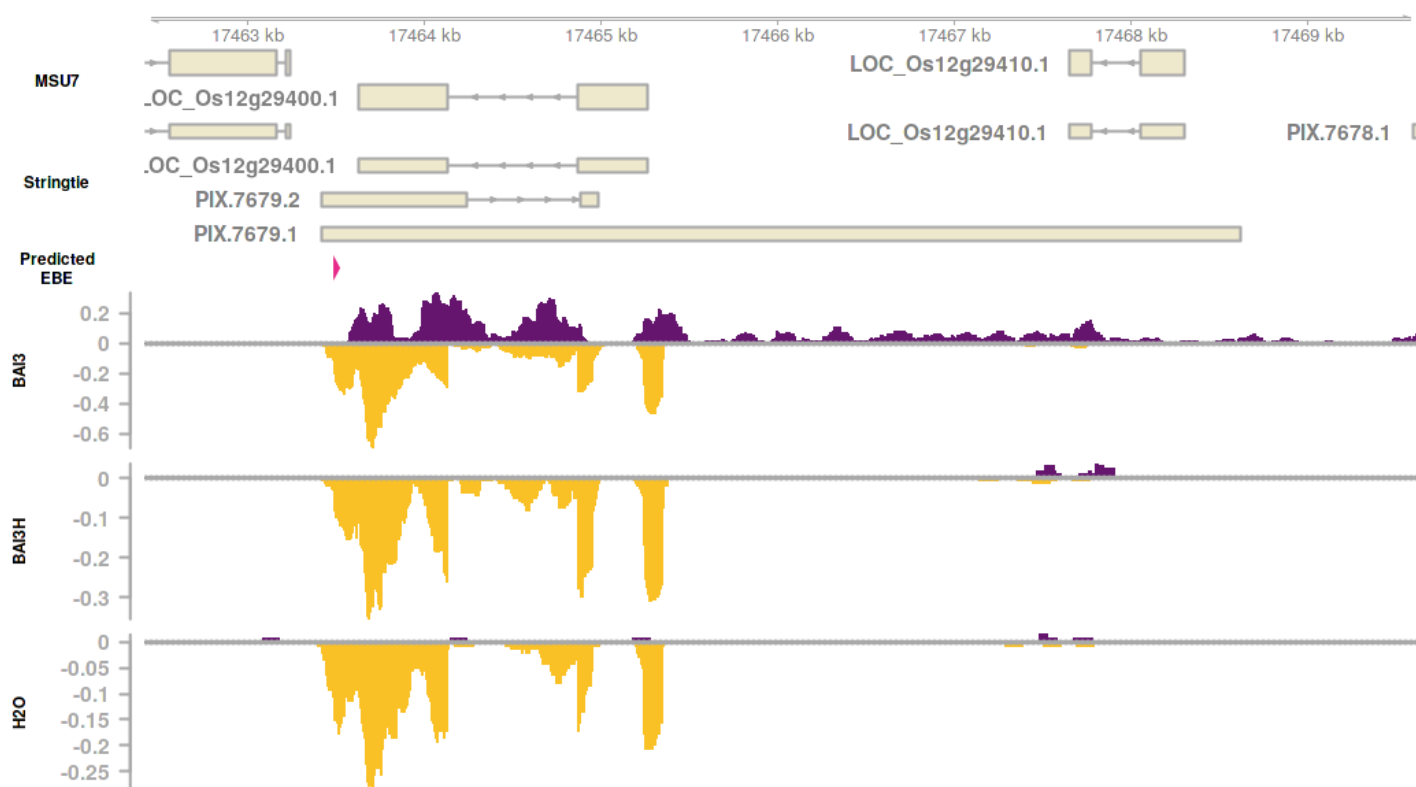

Fig S1 - Page 1

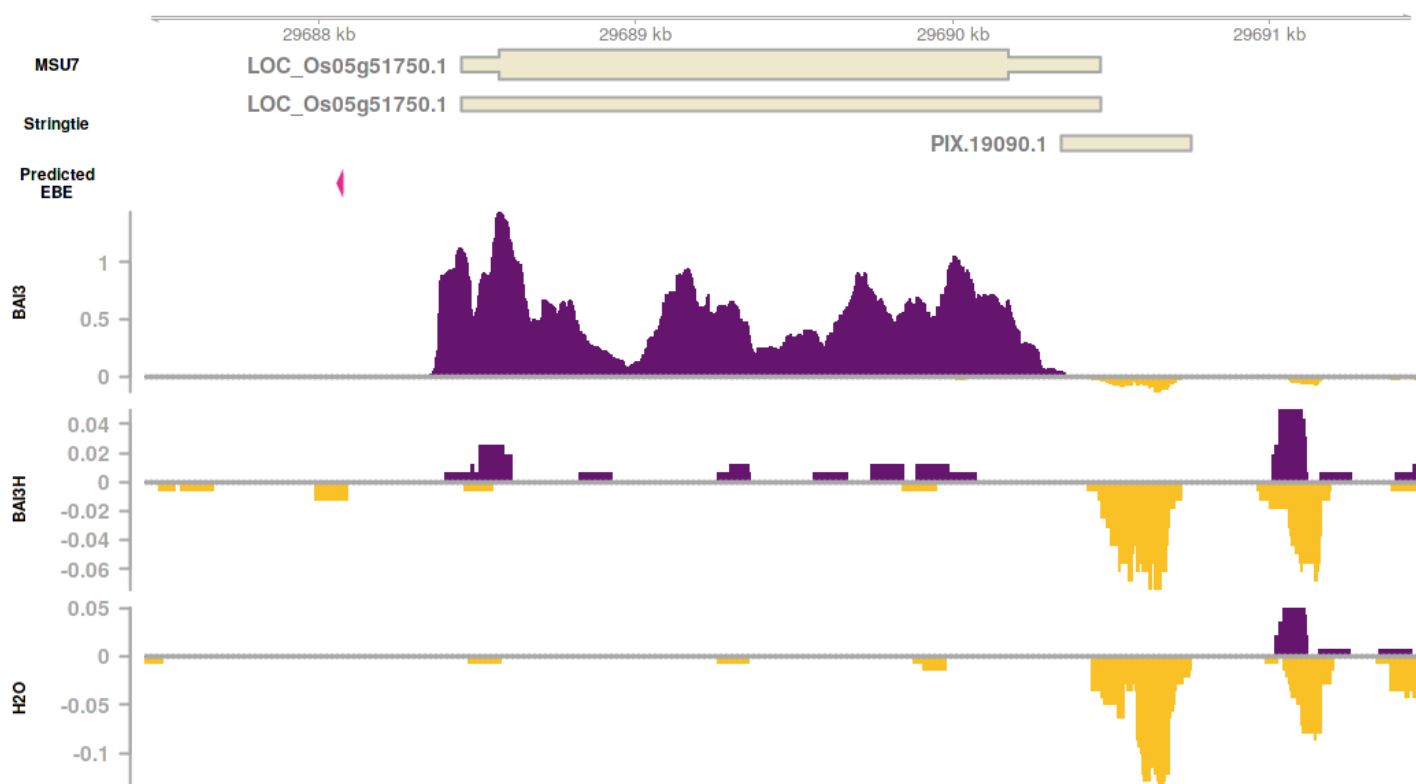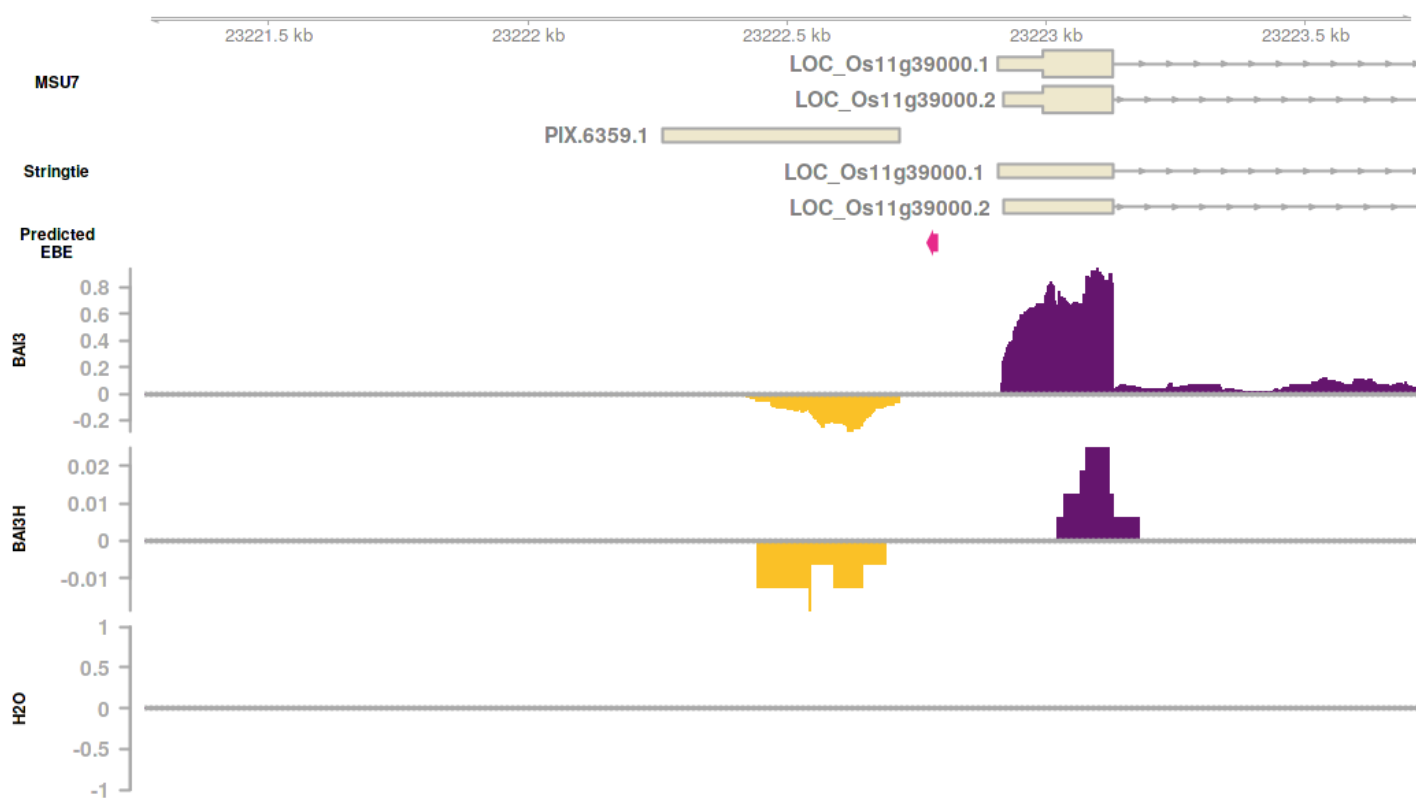

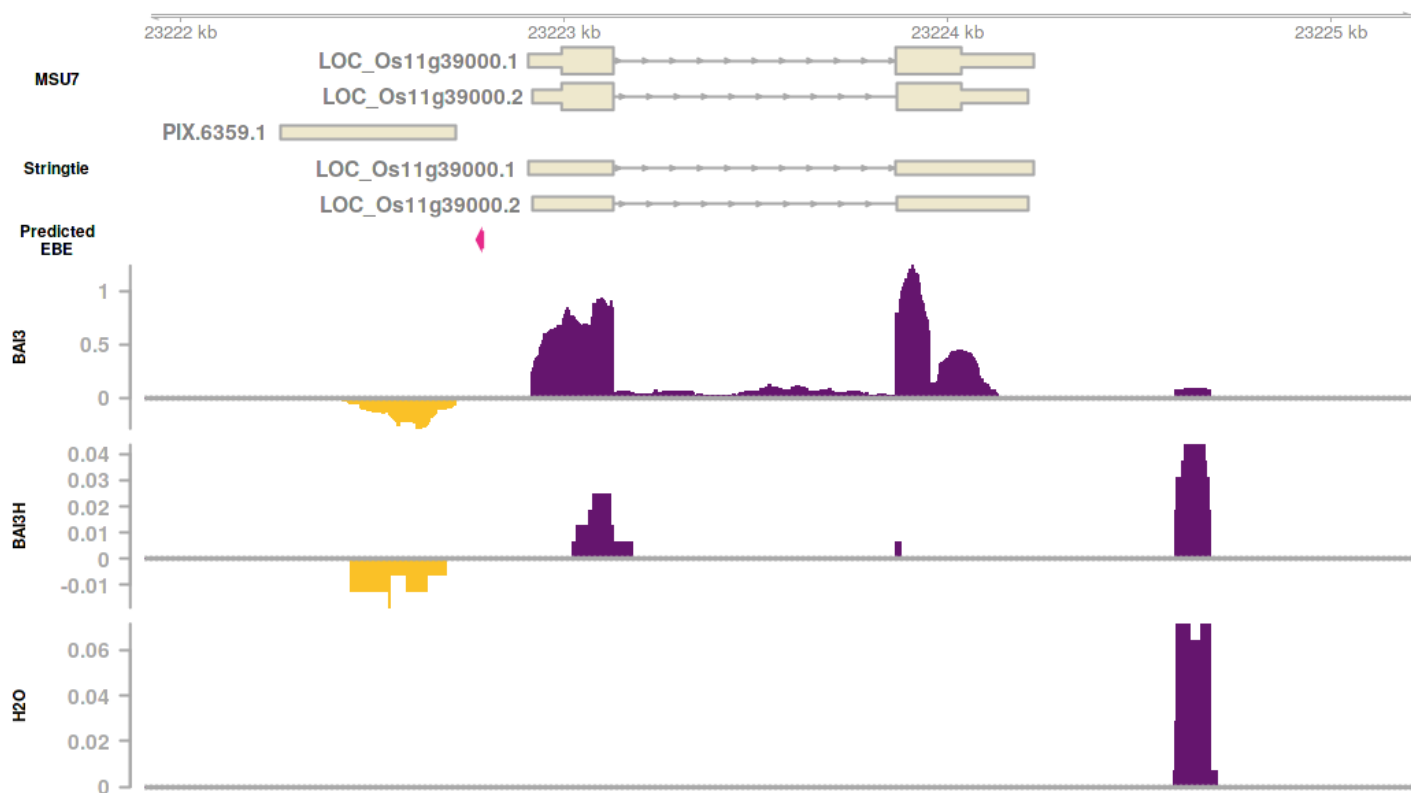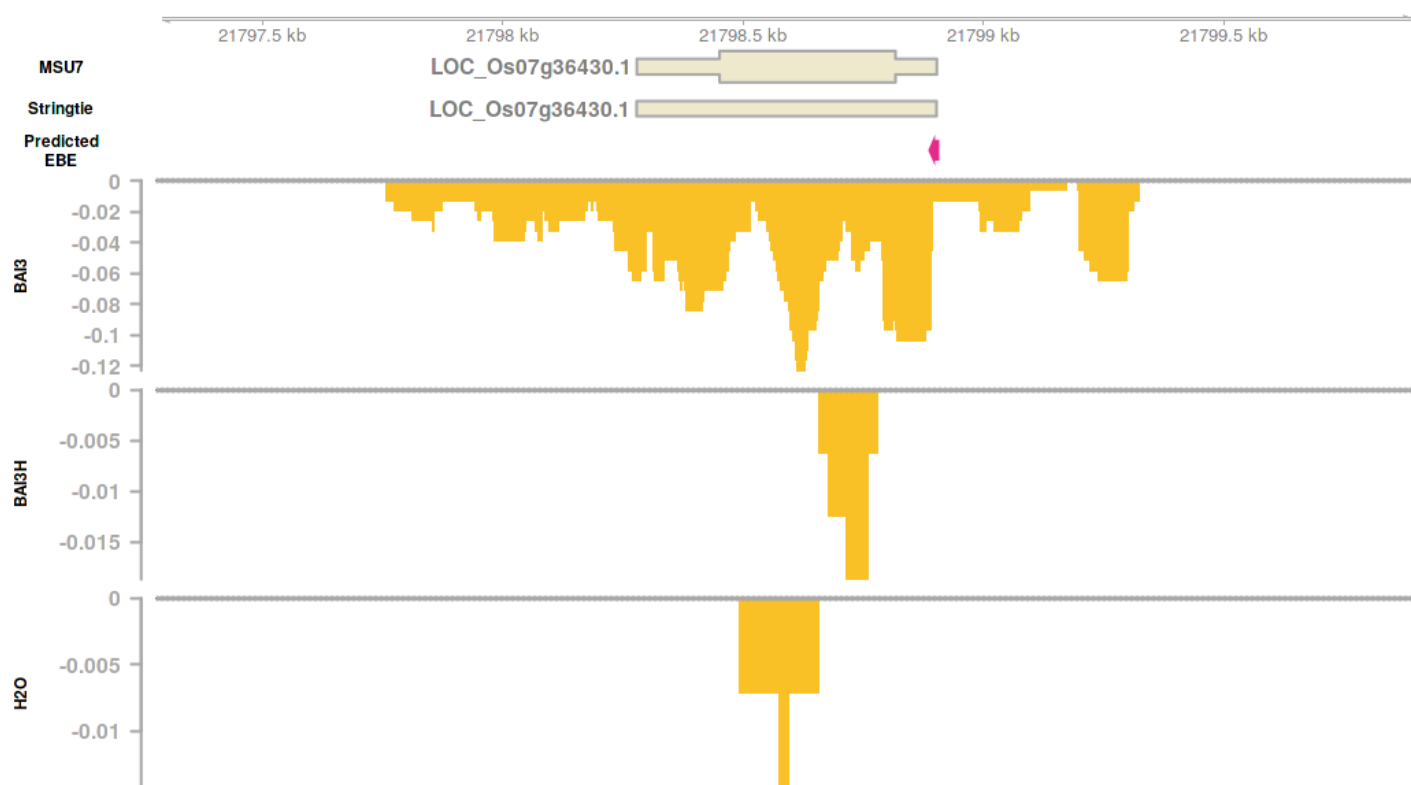

Fig S1 - Page 3

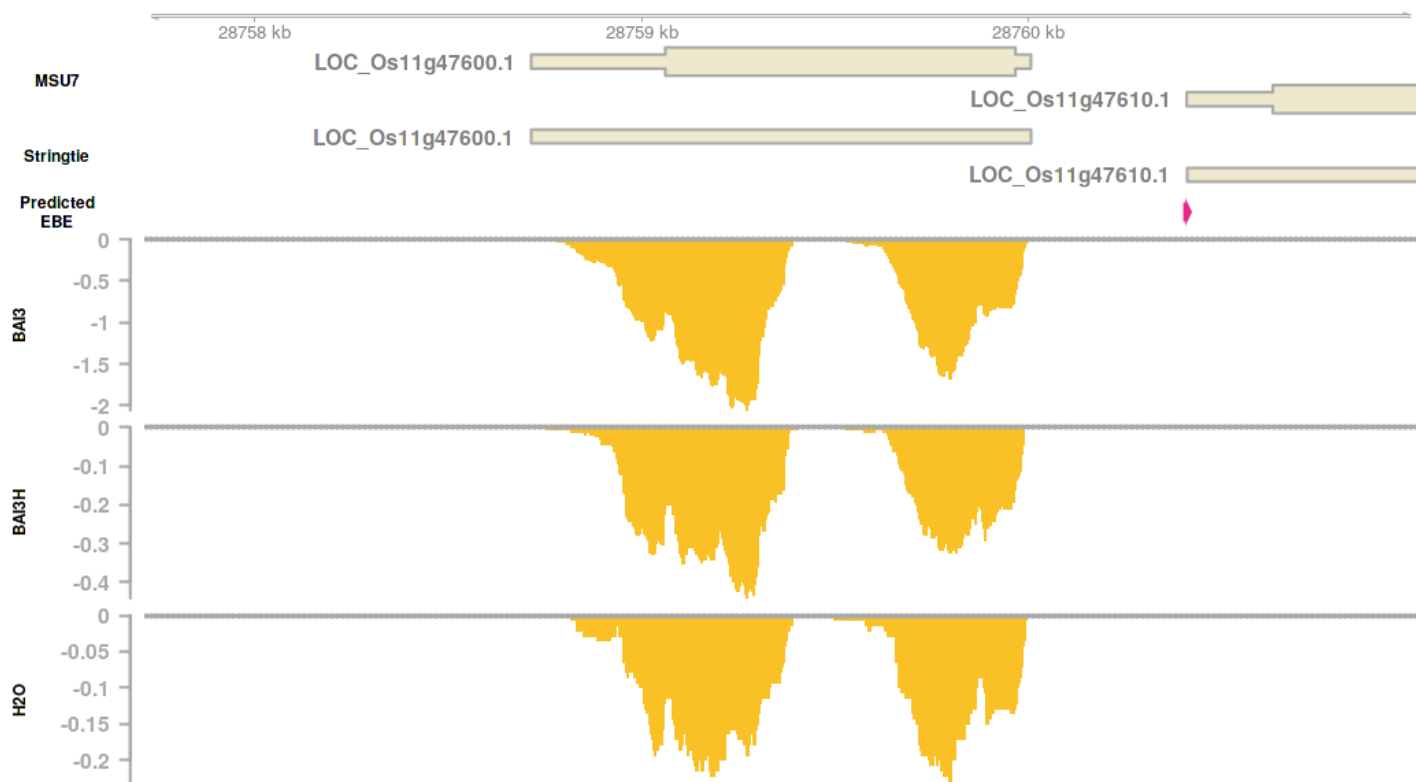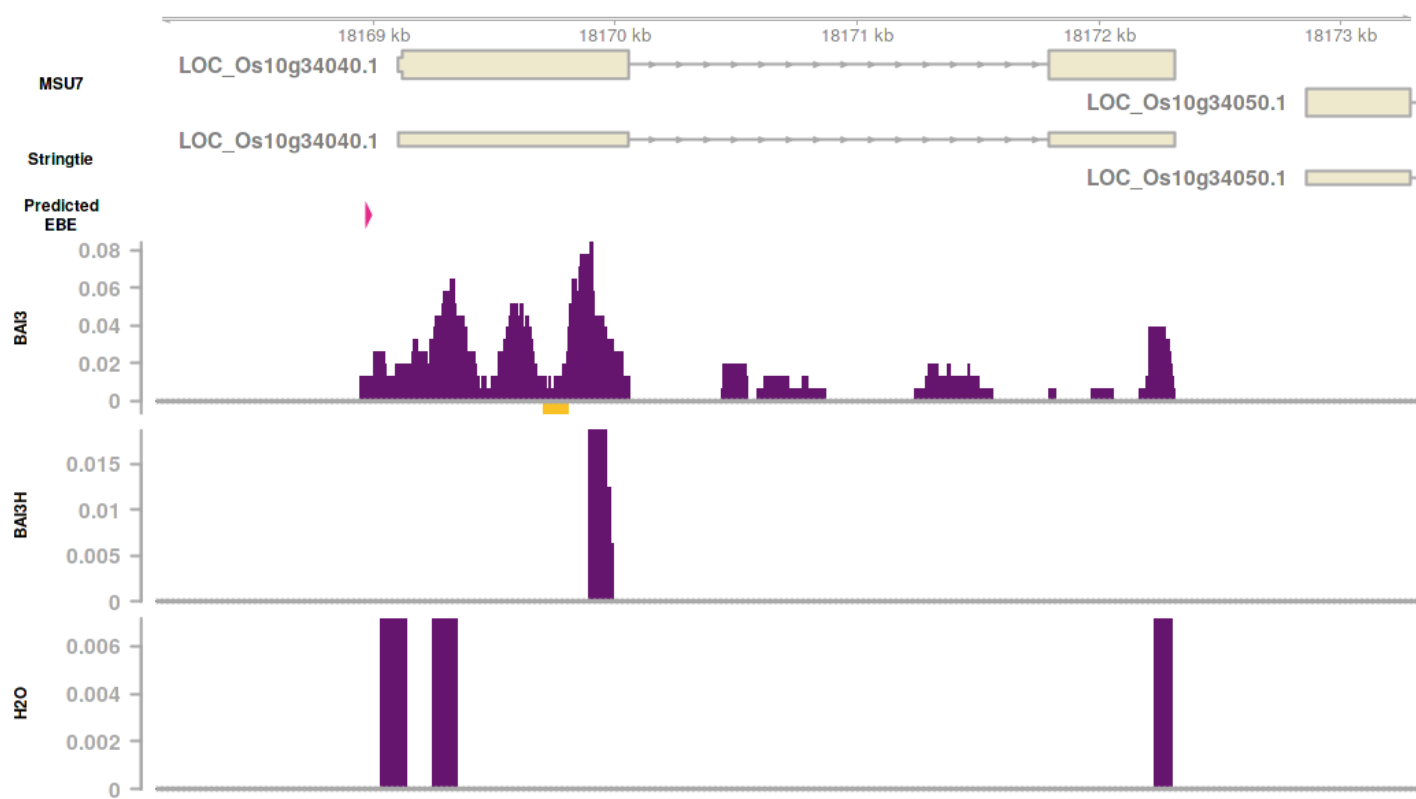

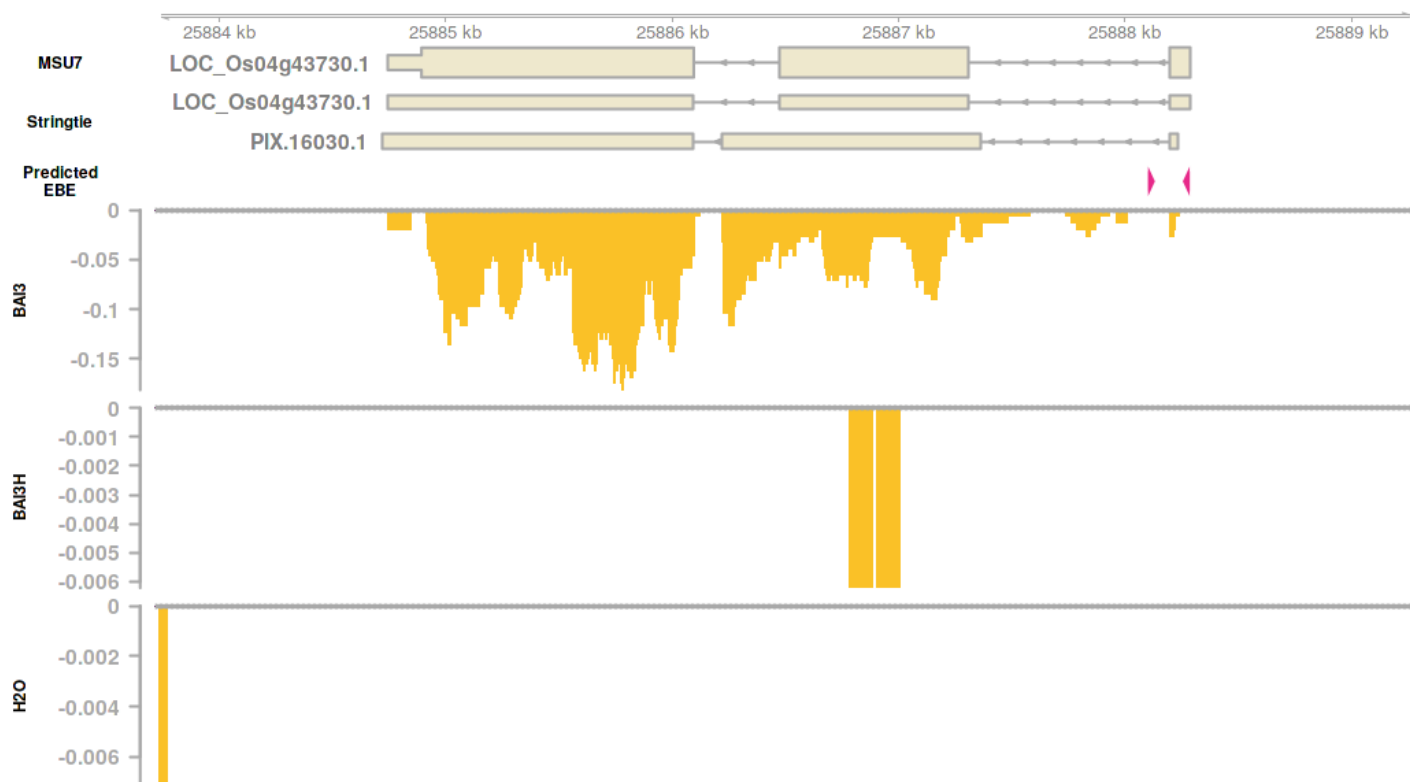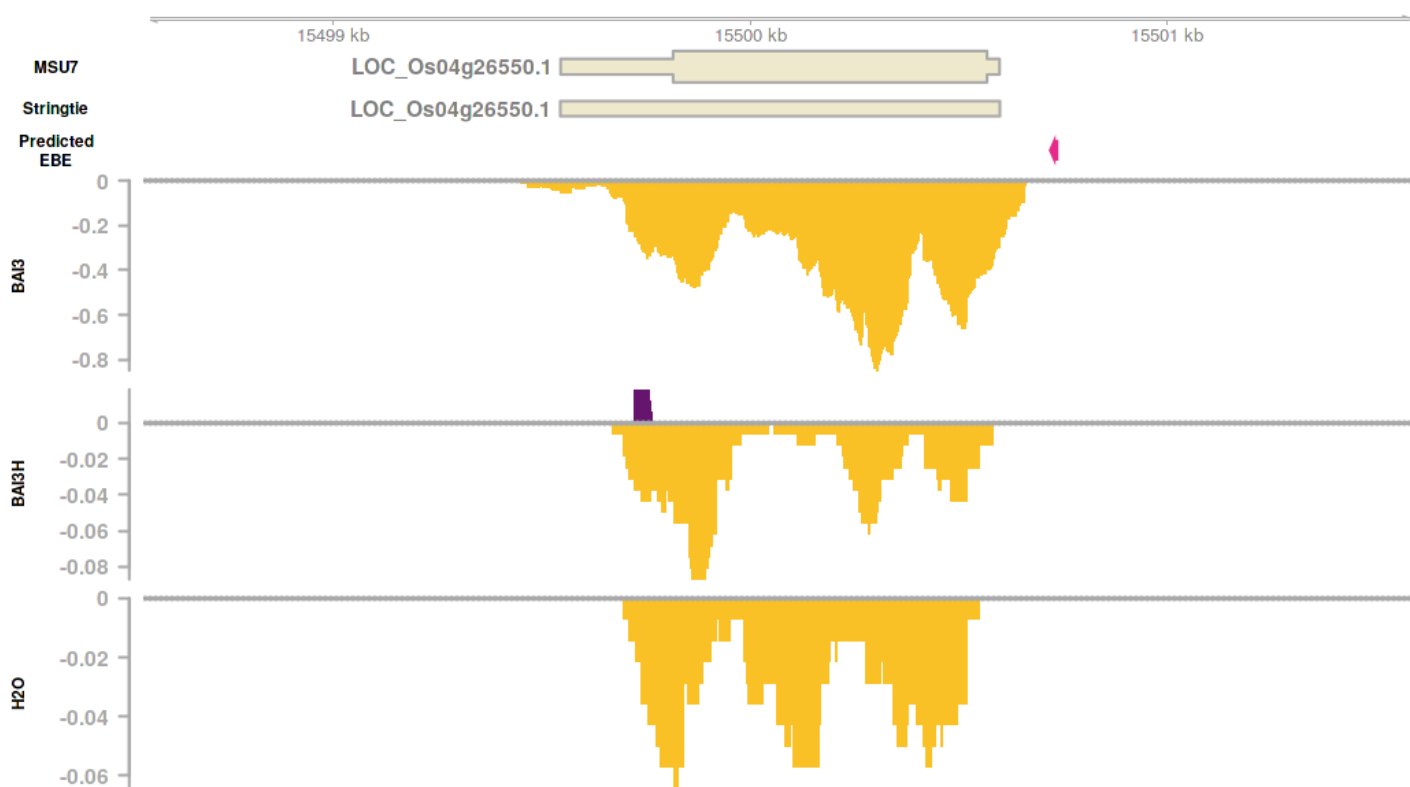

Supplement: Supplementary file 1 [file ijms-23-05559-s001.zip › Fig_S1.pdf]

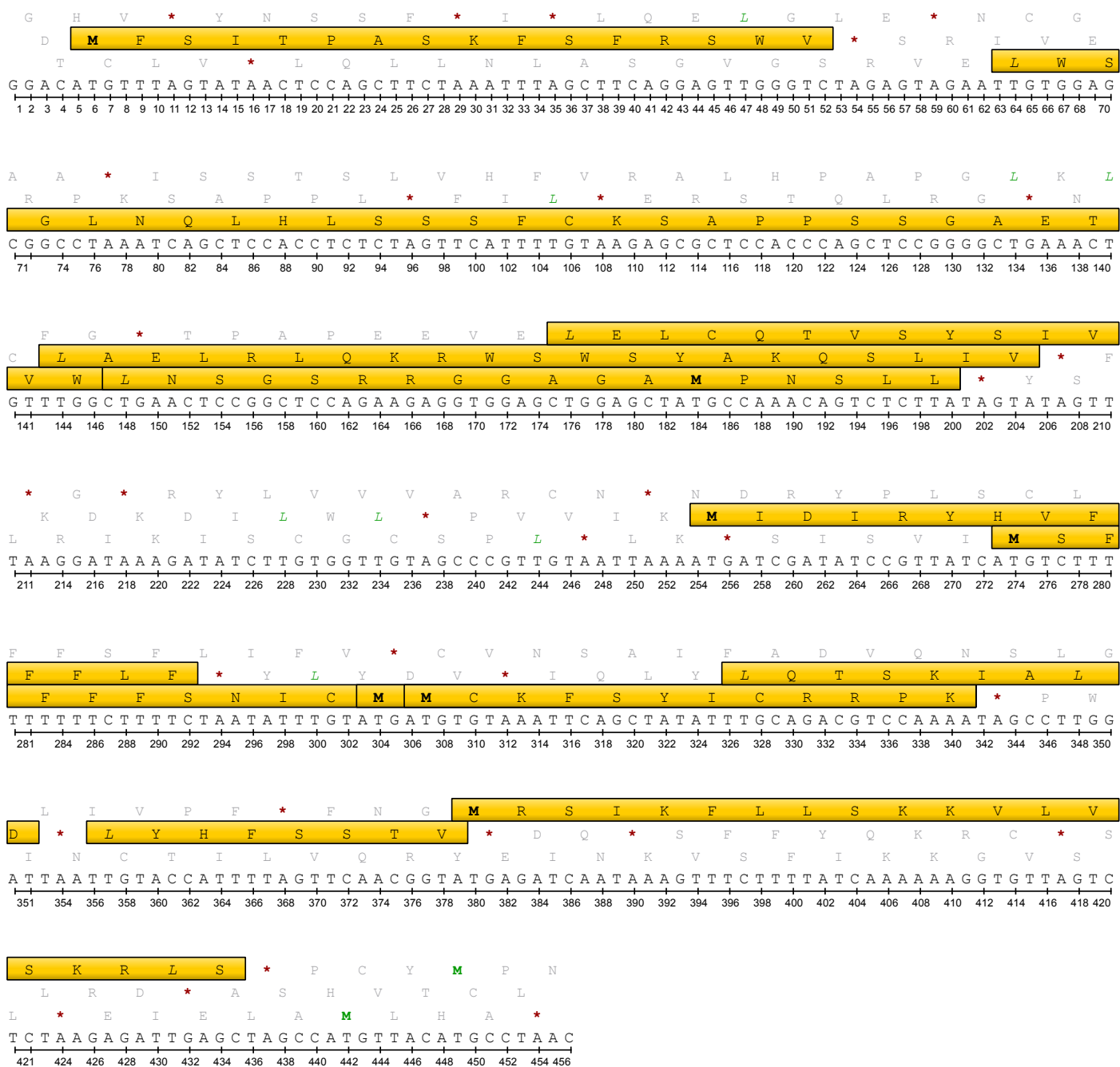

Figure S2

Supplement: Supplementary file 1 [file ijms-23-05559-s001.zip › Fig_S2.pdf]

LOC\_Os11g31190

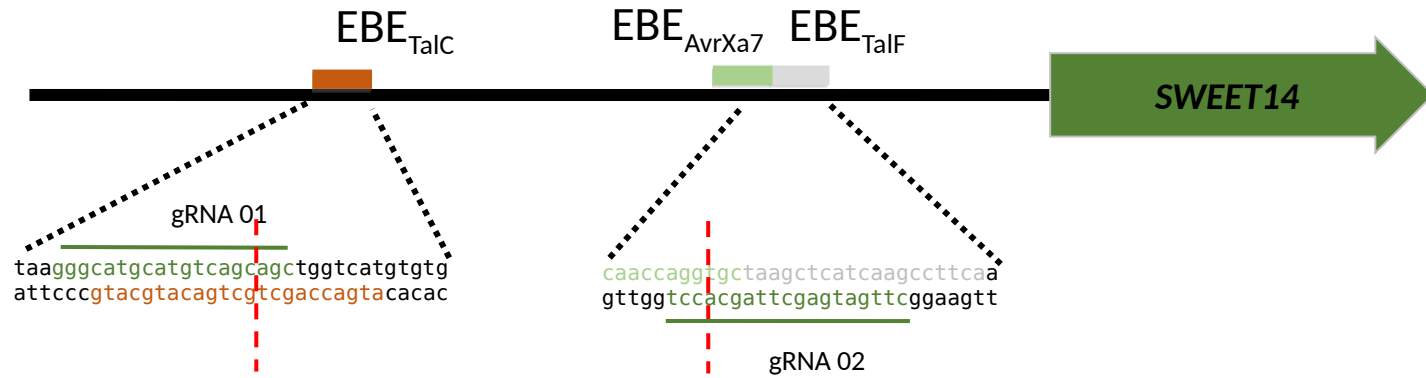

LOC\_Os11g39000

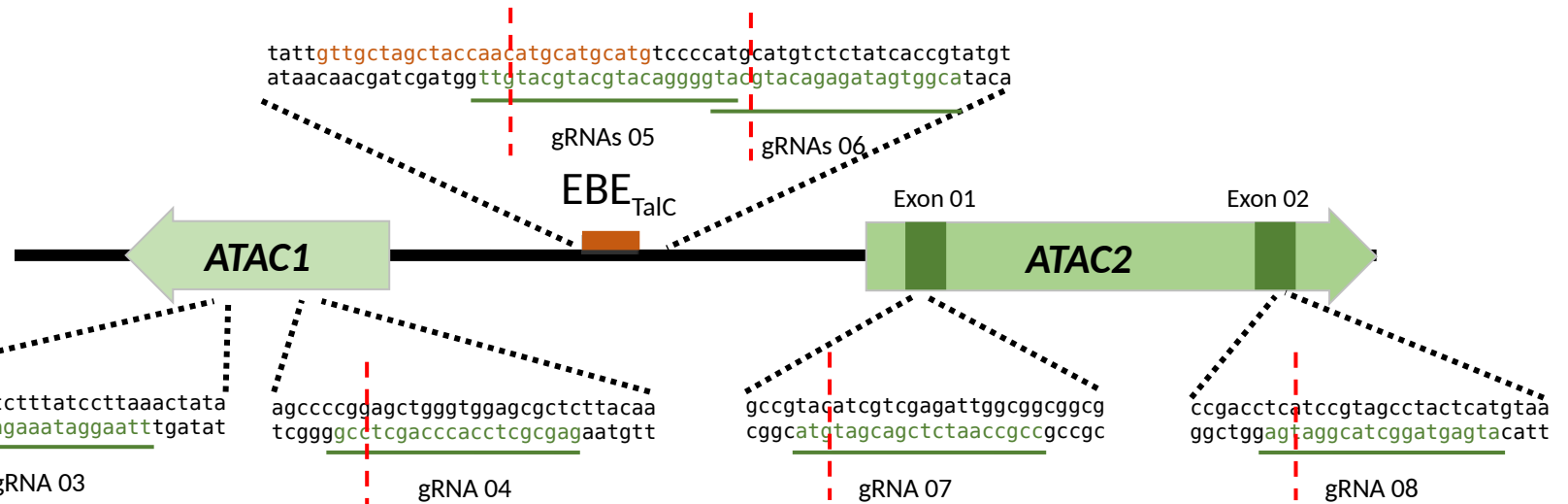

Figure S4

Supplement: Supplementary file 1 [file ijms-23-05559-s001.zip › Fig_S4.pdf]
